# Macrophage targeted iron oxide nanodecoys augment innate immunological and drug killings for more effective *Mycobacterium Tuberculosis* clearance

**DOI:** 10.1186/s12951-023-02103-x

**Published:** 2023-10-10

**Authors:** Ling Shen, Kangsheng Liao, Enzhuo Yang, Fen Yang, Wensen Lin, Jiajun Wang, Shuhao Fan, Xueqin Huang, Lingming Chen, Hongbo Shen, Hua Jin, Yongdui Ruan, Xing Liu, Gucheng Zeng, Jun-Fa Xu, Jiang Pi

**Affiliations:** 1 Department of Microbiology and Immunology, University of Illinois at Chicago, Chicago, IL USA; 2 Guangdong Provincial Key Laboratory of Medical Molecular Diagnostics, The First Dongguan Affiliated Hospital, Guangdong Medical University, Dongguan, China; 3 The Marine Biomedical Research Institute of Guangdong Zhanjiang, The Marine Biomedical Research Institute of Guangdong Medical University, ZhanJiang, Guangdong China; 4 Institute of Laboratory Medicine, School of Medical Technology, Guangdong Medical University, Dongguan, China; 5 Clinic and Research Center of Tuberculosis, Shanghai Key Lab of Tuberculosis, Shanghai Pulmonary Hospital, Tongji University School of Medicine, Shanghai, China; 6 Key Laboratory of Animal Disease Diagnostics and Immunology, Ministry of Agriculture, MOE International Joint Collaborative Research Laboratory for Animal Health & Food Safety, College of Veterinary Medicine, Nanjing Agricultural University, Nanjing, China; 7 Department of Microbiology, Zhongshan School of Medicine, Key Laboratory for Tropical Diseases Control of the Ministry of Education, Sun Yat-Sen University, Guangzhou, Guangdong China

**Keywords:** Nanodecoy, Macrophage-targeting, Tuberculosis, Anti-microbial immunity, Synergistic mtb killing

## Abstract

Tuberculosis (TB), caused by *Mycobacterium tuberculosis* (Mtb) infection, is still one of the top killers worldwide among infectious diseases. The escape of Mtb from immunological clearance and the low targeting effects of anti-TB drugs remain the substantial challenges for TB control. Iron is particularly required for Mtb growth but also toxic for Mtb in high dosages, which makes iron an ideal toxic decoy for the ‘iron-tropic’ Mtb. Here, a macrophage-targeted iron oxide nanoparticles (IONPs)-derived IONPs-PAA-PEG-MAN nanodecoy is designed to augment innate immunological and drug killings against intracellular Mtb. IONPs-PAA-PEG-MAN nanodecoy exhibits preferential uptake in macrophages to significantly increase drug uptake with sustained high drug contents in host cells. Moreover, it can serve as a specific nanodecoy for the ‘iron-tropic’ Mtb to realize the localization of Mtb contained phagosomes surrounding the drug encapsulated nanodecoys and co-localization of Mtb with the drug encapsulated nanodecoys in lysosomes, where the incorporated rifampicin (Rif) can be readily released under acidic lysosomal condition for enhanced Mtb killing. This drug encapsulated nanodecoy can also polarize Mtb infected macrophages into anti-mycobacterial M1 phenotype and enhance M1 macrophage associated pro-inflammatory cytokine (TNF-α) production to trigger innate immunological responses against Mtb. Collectively, Rif@IONPs-PAA-PEG-MAN nanodecoy can synergistically enhance the killing efficiency of intracellular Mtb in in vitro macrophages and ex vivo monocyte-derived macrophages, and also significantly reduce the mycobacterial burdens in the lung of infected mice with alleviated pathology. These results indicate that Rif@IONPs-PAA-PEG-MAN nanodecoy may have a potential for the development of more effective therapeutic strategy against TB by manipulating augmented innate immunity and drug killings.

**Graphic Abstract:**

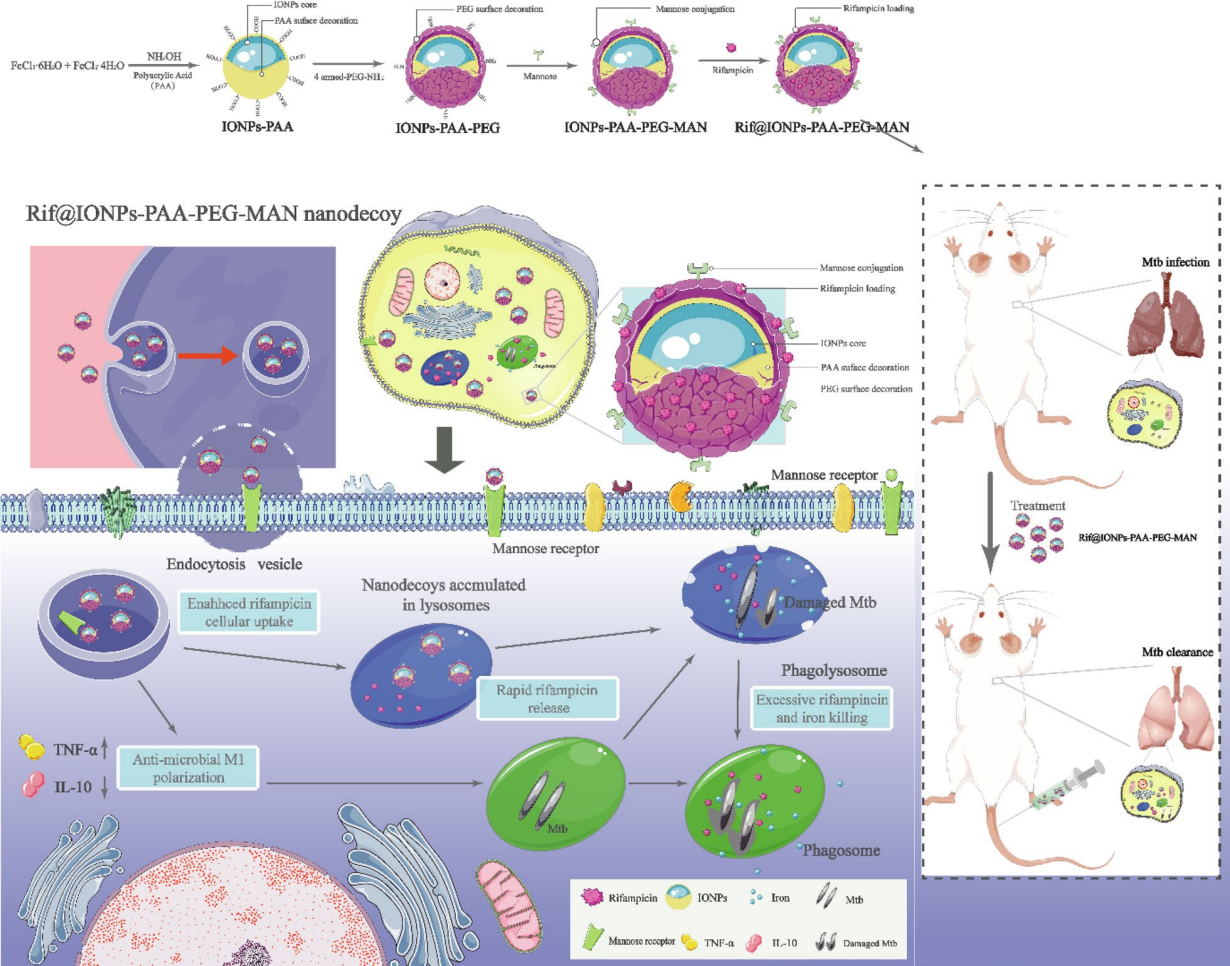

**Supplementary Information:**

The online version contains supplementary material available at 10.1186/s12951-023-02103-x.

## Background

Despite global countermeasure efforts, tuberculosis (TB) remains one of the top killers worldwide among infectious diseases, with 10.6 million new cases and 1.6 million mortalities reported in 2021 [1]. Although *Mycobacterium tuberculosis* (Mtb) infects both HIV (human immunodeficiency virus)-positive and negative individuals, the risk of progression to active TB in those co-infected with Mtb (latent TB) and HIV is significantly increased, which makes HIV-positive TB one of the most risking issues for TB. Based on the risks of HIV and Mtb co-infection, the estimated 1.6 million global TB deaths in 2021 include 1.4 million HIV negative and 0.2 million HIV positive deaths [1]. Mtb, the tricky pathogen causing TB, is unanimously recognized as one of the most successful human pathogens due to its strong ability to persist and survive in the macrophages of immunocompetent individuals. Macrophages, as the key immune component of innate immunity and host cell against Mtb, can phagocytose and destroy different pathogens, and initiate protective adaptive immune responses via antigen presentation to T cells. However, Mtb possess a plethora of complex strategies to evade the major anti-microbial mechanisms of host cells, such as the inhibition of autophagy/apoptosis and the escape of lysosome-mediated killings [2–4], which ultimately contribute to the development of latent or active TB. The design of novel methods to inhibit these immunological escape ways of Mtb would benefit the development of more effective anti-TB strategies.

Some potent antibiotics against Mtb, such as rifampicin and isoniazid, have remarkably reduced the death of TB in the past decades. However, the poor targeting effects of these anti-TB drugs lead to the poor macrophage cellular uptake for Mtb killings. The internalized anti-TB drugs in macrophages would also be rapidly eliminated due to the drug efflux pump and drug metabolism of eukaryocytes [5, 6], which lead to very low intracellular drug contents. Moreover, the internalized drugs can’t be accumulated into the Mtb hided subcellular spaces for direct killing of intracellular stubborn Mtb, which further restricts the efficiency of anti-TB drugs against intracellular Mtb. These cellular and subcellular restrictions for the low drug efficiency of anti-TB drugs finally results in a long-term therapy with combined drugs from 6 to 12 months. The prolonged treatment period would introduce very heavy burden to patients that induces liver and kidney injury and also amplify the drug non-compliance to develop drug resistant TB [7, 8], which leads to the current drug-resistant TB epidemics and high fatality. Thus, there is a desperate need to diversify new approaches to improve the existing therapy regimen in terms of better efficacy.

Functional nanomaterials are capable of accumulating drugs at target cells and regions [9, 10], thus providing new possibilities for selective delivery of antibiotics into Mtb infected macrophages and tissues [11]. Such nanoscale strategy shows the potential to achieve higher intracellular drug concentration locally while limiting systemic toxicities, optimizing drug dosage, improving efficacy, shortening therapy duration and minimizing side effects. Moreover, designed nanomaterials targeting different intracellular spaces [12–14] can offer the convenience to deliver anti-TB drugs into the Mtb hided subcellular spaces, which is therefore expected to kill the intracellular stubborn Mtb more effectively. We have recently conceptualized that macrophage-targeted selenium nanoparticles could be served as potential anti-TB system for enhanced host cell phagolysosomal destruction and anti-TB drugs for more effective Mtb killings [15]. However, the innate immunity enhancement effects and biocompability of the reported selenium nanoparticles remain to be further improved. Thus, the development of more functional nanomaterials that augmenting the innate immunity and drug killing with better biocompatibility may finally benefit TB therapy.

While playing important roles in many essential biological processes, iron is also essential for the survival/growth of Mtb and other microorganisms. In macrophages, phagocyted Mtb could encounter extremely low free iron levels if fused into lysosomes [16], for which Mtb keeps trying their best for iron acquisition from other subcellular structures [17]. However, the extreme doses of iron are also toxic to microorganisms by disturbing their genetic material, normal metabolism or inhibiting their immune escape [18–20]. Iron oxide nanoparticles have been proposed as a kind of agents with magnetically positionable anti-tumor and anti-microbial properties [21, 22]. Leidinger et al. have demonstrated that iron oxide nanocontainers with isoniazid inside can function as “Trojan Horses” and show efficient, active uptake into both Mtb infected macrophages and even into mycobacterial cells [23], which indicated the strong possibility of iron oxide as anti-TB drug delivery systems. More interestingly, cancer and pharmacokinetics studies show that iron oxide nanoparticles can also induce pro-inflammatory macrophage polarization to potentiate macrophage- modulating immunotherapy strategies [24–26]. Importantly, iron oxide nanoparticles can readily permeabilize lysosomal membrane for lysosome accumulation [27], making it possible to utilize iron oxide nanoparticles as a kind of potential nanodecoys to attract Mtb nearby for more effective killing with augmented innate immunity.

Here, combining our decades-long TB immunology expertise [28–31] and nanotechnology advantages [32–37] we developed an iron oxide-dependent nanodecoy that augmenting drug and innate immunity killings of intracellular Mtb. This iron oxide nanoparticle with polyacrylic acid (PAA)/polyethylene glycol (PEG) coating and mannose functionalization, namely IONPs-PAA-PEG-MAN, were constructed to act as macrophage- targeted drug delivery system and a kind of novel nanodecoy aiming to the ‘iron-tropic’ property of Mtb for more effective Mtb killings. The proposed Rif@IONPs-PAA-PEG-MAN synergistically enhanced the killing effects of intracellular Mtb in macrophages and reduced the Mtb burdens in the lung of mice, which might have a potential for better therapeutic strategy against TB and drug-resistant TB.

## Materials and methods

### Preparation and characterization of Rif@IONPs-PAA-PEG-MAN nanodecoy

Polyacrylic acid (PAA) protected iron oxide nanoparticles were prepared using a previously reported method [38]. Briefly, an iron salt solution containing 0.62 g of Iron (III) chloride hexahydrate (Sigma, USA) and 0.32 g of Iron (II) chloride tetrahydrate (Sigma, USA) dissolved 2 ml water with 100 µl of 12 N hydrochloric acid (Sigma, USA) was drop-wise added into 16.8 ml N_2_-purged deionized water with 1.8 ml of 30% ammonium hydroxide (Sigma, USA) solution under vigorous stirring. After 30 s of the addition of iron salt solution, a PAA (Sigma, USA) solution (820 mg) was added into the mixture for 1 h stirring. The resulting suspension was centrifuged at 4 000 rpm for 30 min to collect the supernatant, which was further washed with deionized water using ultra-filtration tubes (~ 30 k) at 4000 rpm to obtain IONPs-PAA. 2 ml EDC·HCl (1-(3-Dimethylaminopropyl)-3-ethylcarbodiimide hydrochloride, Sigma, USA) solution (10 mg/ml) was added into 20 ml IONPs-PAA (10 mg/ml) under stirring for 10 min, followed by the addition of 10 mg of 4 armed-PEG (polyethylene glycol)-NH_2_ (Sigma, USA) in 1 ml water for overnight stirring. The obtained nanoparticles were collected and washed with deionized water using ultra-filtration tubes (~ 30 k) to obtain IONPs-PAA-PEG. D-mannose (100 mM, Sigma, USA)) dissolved in sodium acetate (pH 4.0; 0.1 M, Sigma, USA) were subsequently added to IONPs-PAA-PEG solution under stirring for mannosylation using a similar method [35, 39]. Mannosylated nanoparticles were further washed with deionized water using ultra-filtration tubes to obtain IONPs-PAA-PEG-MAN. 0.5 ml rifampicin (Rif, Sigma, USA) solution (5 mg/ml in methanol) was added into 2 ml IONPs-PAA-PEG-MAN solution (5 mg/ml) for overnight incubation, followed by dialysis (MWCO: 8000–14,000) against deionized water to obtain Rif@IONPs-PAA-PEG-MAN. 3,3’-Diethylthiadi-carbocyanine iodide (DI, Sigma, USA) or coumarin-6 (C6, Sigma, USA) were used to prepare DI@IONPs-PAA-PEG-MAN or C6@ IONPs-PAA-PEG-MAN using the similar method. IONPs-PAA-PEG-MAN and Rif@IONPs-PAA-PEG-MAN were characterized by TEM (Philips, Holland), FTIR (Bruker, German), UV-Vis (Agilent, USA), DLS (Malvern Instruments, UK), XRD (Bruker, German) and XPS (Thermo Scientific, USA).

### Bacteria and cell culture

BCG (Bacillus Calmette-Guerin) and H37Rv were cultured in 7H9 medium supplied with 10% OADC. All experiments about BCG were performed at BSL (biosafety level)-2 lab and all experiments about H37Rv were performed at BSL (biosafety level)-3 lab. THP-1 cells, A549 cells, and murine RAW264.7 cells were cultured with RPMI 1640 medium supplemented with 10% FBS (Fetal bovine serum) in a humidified atmosphere of 5% CO_2_ at 37 ℃. Human lung microvascular endothelial cells (HLMVEC) were cultured in EBM-2 medium supplemented with EGM-2-MV in a humidified atmosphere of 5% CO_2_ at 37 ℃.

### Cellular viability analysis

THP-1 cells, A549 cells, HLMVEC cells and RAW264.7 cells were seeded into 96 well plates with a density of 1 × 10^4^ cells/well for 24 h (THP-1 cells were stimulated with 100 nM of PMA). Cells were then incubated with IONPs-PAA-PEG-MAN for 72 h. After that, 3-(4,5-Dimethylthiazol-2-yl)-2,5-diphenyltetrazolium bromide (MTT, 10 µl, 5 mg/mL, Sigma, USA) were then added for 4 h incubation. After medium was removed, the cells were suspended in 150 µl of DMSO (dimethylsulfoxide) for 15 min shaking, followed by spectrophotometer (TECAN, Switzerland) analysis at 570 nm.

### Cellular uptake and mechanism analysis of IONPs-PAA-PEG-MAN in macrophages

The cellular uptake of IONPs-PAA-PEG-MAN in THP-1 cells and HLMVEC cells were analyzed by detecting the fluorescence of C6@IONPs-PAA-PEG-MAN. Cells were seeded into 96 well plates with a density of 1 × 10^4^ cells/well for 24 h (THP-1 cells were stimulated with 100 nM PMA (phorbol myristate acetate)) and incubated with C6@IONPs-PAA- PEG-MAN for designed times. After washed with PBS (phosphate buffer) and lysed with 0.5% Triton X-100 in 0.2 M NaOH (sodium hydroxide) solution, microplate reader was used to measure the fluorescence intensity inside the wells with excitation and emission wavelengths set at 485 and 528 nm, respectively. Standard curves for were constructed by suspending C6@IONPs-PAA-PEG-MAN in a similar way as cell sample preparation, which showed R^2^ = 0.9869. Cellular uptake of C6@IONPs-PAA-PEG in THP-1 cells were also measured using the same method, with R^2^ = 0.9987 for the standard curves of C6@ IONPs-PAA-PEG. The uptake of nanoparticles by cells was calculated from the standard curve and expressed as the amounts of nanoparticles (µg) taken up per 10^6^ cells.

Cellular uptake mechanism of IONPs-PAA-PEG-MAN in macrophage was determined under different uptake inhibition conditions. THP-1 cells were seeded into 96 well plates with 100 nM PMA stimulation in a density of 1 × 10^4^ cells/well for 24 h, and then pre-treated with different inhibitors for 1 h, except that nystatin was pre-treated for 30 min. Final concentration of specific inhibitors were listed as following: sodium azide (NaN_3_) 10 mM, sucrose 0.45 M, 2-deoxy-Dglucose (DOG) 50 mM, 5-(N-Ethyl-N-isopropyl)amiloride (5-EIPA) 60 µM, nystatin 10 µg/mL and wortannin 1ug/ml. Then cells were further incubated with 10 µg/ml C6@IONPs-PAA-PEG-MAN for 3 h and the control cells were incubated with 10 µg/ml C6@IONPs-PAA-PEG-MAN without any inhibitions. For investigation of energy-dependent pathways, cells were treated in medium at 4 ℃ for 4 h, followed by C6@IONPs-PAA-PEG- MAN treatment for 3 h. For mannose competition assay, mannose were added to cells for 1 h incubation, followed by C6@IONPs-PAA-PEG-MAN treatment for 3 h. After washed with PBS and lysed with 0.5% Triton X-100 in 0.2 M NaOH solution, microplate reader was used to measure the fluorescence intensity inside the wells with excitation and emission wavelengths set at 485 and 528 nm, respectively. The cellular uptake efficacy was expressed as the percentage of the fluorescence of the testing wells over that of the control wells.

### Cellular uptake of IONPs-PAA-PEG-MAN and IONPs-PAA-PEG in macrophages by iron element analysis

The cellular uptake of IONPs-PAA-PEG-MAN and IONPs-PAA-PEG in THP-1 cells were also analyzed by ICP-AES using a similar method as previously described for metal ion analysis [40]. The cells were seeded at a density of 1 × 10^6^ into 6 well plates with 100 nM PMA stimulation for 24 h. Cells were incubated with 100 µg/ml of IONPs-PAA-PEG-MAN or 100 µg/ml of IONPs-PAA-PEG for 1 h. After washed with PBS, cells were collected for microwave digestion. Then, the iron element (Fe) were analyzed by ICP-AES (Agilent, USA).

### Intracellular localization of IONPs-PAA-PEG-MAN in macrophages

Intracellular localization of IONPs-PAA-PEG-MAN in THP-1 cells was investigated by fluorescence microscopy with specific staining of lysosomes. The cells were seeded at a density of 5 × 10^5^ into confocal dishes with 100 nM PMA stimulation for 24 h, cells were incubated with 10 µg/ml of C6@IONPs-PAA-PEG-MAN for various periods of time. After that, cells were incubated with lysotracker red for 30 min, followed by confocal microscopy (Zeiss, German) analysis after PBS wash.

### In Vitro Drug Release behaviors of Rif@IONPs-PAA-PEG-MAN nanodecoy

An aliquot of 40 mg of the Rif@IONPs-PAA-PEG-MAN were put into a dialysis bag (MWCO: 8000–14,000). The dialysis bag (MWCO: 8000–14,000) was then put into 10 ml of PBS solution at different pH (7.4 or 5.5) with constant shaking at 37 ℃ in a tube. At predetermined time intervals, 200 µl of solution was taken out from the vial (outside the dialysis bag) with pipet and the same volume of fresh PBS solution was added. The released rifampicin was measured by high performance liquid chromatography (HPLC, Agilent, Santa Clara, CA) equipped with a Luna C18 column (250 mm × 4.6 mm × 5 μm) and wavelength of 480 nm using 25 mM ammonium acetate/acetonitrile (45%/55%) as the mobile phase.

### Ex Vivo **Cellular Uptake of IONPs-PAA-PEG-MAN by T cells, B cells, endothelium and macrophages in intraepithelial lymphocytes (IEL) of macaques**

Intestine from Mtb infected rhesus macaques was used for IEL isolation in BSL-3 lab, all animal experimental procedures and protocols were approved by the University of Illinois Chicago Animal Care Committee. Purified IEL cells were seeded into 96 well plates with a density of 8 × 10^5^ cells/well. Then, 10 µg/ml of DI@IONPs-PAA-PEG-MAN or DI@IONPs-PAA-PEG were added into the cells for 3 h incubation. After that, cells were collected, washed with PBS containing 2%FBS and 2 mM EDTA (Ethylene Diamine Tetraacetic Acid), and then stained with PerCP anti-human CD14 antibody (Biolegend, USA), PB anti-human CD3 antibody (Biolegend, USA) and FITC anti-human CD20 antibody (Biolegend, USA) at room temperature for 30 min. After washed with PBS containing 2% FBS and 2 mM EDTA, cellular uptake by T cells, B cells, endothelium and macrophages were analyzed by flow cytometry (BD) after fixation by 4% formalin. Endothelium was gated in the cell population at the top right corner. CD3, CD20 and CD14 cells were gated in the mucosal immune cells. Fluorescence positive cells were gated to determine the cellular uptake in different cells.

### Biological TEM analysis of Rif@IONPs-PAA-PEG-MAN nanodecoy in Mtb infected macrophages

THP-1 cells were seeded at a density of 1 × 10^6^ into 6 plates with 100 nM PMA stimulation for 24 h, and then infected with H37Rv (4 h infection) using MOI (multiplicity of infection) = 1. The infected cells were treated with IONPs-PAA-PEG-MAN or Rif@IONPs-PAA-PEG-MAN for 72 h, and then collected, washed with PBS and fixed by 2.5% glutaraldehyde and 2% paraformaldehyde for 48 h at 4 ℃. The fixed cell samples were washed with PBS and then further fixed with 0.1% osmic acid for 2 h. After washed with PBS, the samples were dehydrated with sequential treatment of 50%, 70%, 85%, 90%, and 100% ethanol, respectively. Then, the samples were embedded in resin, cut into ultrathin slices, stained with 2% uranyl acetate and 0.2% lead citrate before TEM (JEOL, Japan) observation.

### Effects of Rif@IONPs-PAA-PEG-MAN nanodecoy on the polarization of Mtb infected macrophages

THP-1 cells were seeded at a density of 1 × 10^6^ into 6 plates with 100 nM PMA stimulation for 24 h, and then infected with BCG (24 h infection) or H37Rv (4 h infection) using MOI = 1. After washed with PBS, cells were treated with IONPs-PAA-PEG-MAN, Rif@IONPs-PAA-PEG-MAN or rifampicin for 72 h. Supernatants were collected by centrifugation for nitrite concentration analysis following manufacturer’s protocol. The collected cells were incubated with APC anti-human CD11b antibody (Biolegend, USA), PerCP anti-human CD14 antibody (Biolegend, USA), Alexa Fluor 700 anti-human CD206 antibody (Biolegend, USA) and PE anti-human CD80 antibody (Biolegend, USA) for 30 min at 4 ℃. After washed with FBS-EDTA-PBS, cells were fixed by PBS containing 2% formalin before analysis by a flow cytometry (BD, USA). To determine intracellular TNF-α and IL-10 level, cells were stained with APC anti-human CD11b antibody (Biolegend, USA) and PerCP anti-human CD14 antibody (Biolegend, USA). After washed with PBS, cells were treated with cytofix/cytoperm for 30 min at room temperature, and then incubated with PE anti-human TNF-α antibody (Biolegend, USA) and PE/Cy7 anti-human IL-10 antibody (Biolegend, USA) for 30 min at 4 ℃. After washed with FBS-EDTA-PBS, cells were fixed by PBS containing 2% formalin before analysis by a flow cytometry (BD, USA). The expression of CD80 and CD206, and the intracellular level of TNF-α and IL-10 were all gated in CD11b + CD14 + cells.

### Intracellular rifampicin concentration analysis of Rif@IONPs-PAA-PEG-MAN nanodecoy treated macrophages

Intracellular rifampicin concentration was determined using the similar method as reported [35, 41]. THP-1 cells were seeded at a density of 2 × 10^6^ into 6 well plates with 100 nM PMA stimulation for 48 h, and then treated with 5 µg/ml rifampicin or Rif@IONPs- PAA-PEG-MAN containing 5 µg/ml rifampicin for designed times. After washed with PBS, cells were collected for cell counting, and suspended in 0.2 ml PBS solution to mix with 25 µl sodium dodecyl sulfate (SDS) solution (2%) for 5 min lysis at room temperature. Then, the samples were homogenized for 5 min by ultrasonic bath and mixed with 0.3 ml ammonium acetate (50 mM) plus 25 µl roxithromycin solution (500 ng/ml). Then, samples were extracted with 5 ml methyl tert-butyl ether for 15 min for 2980 g centrifugation of 2 min, the organic layer was then separated, mixed with 50 µl butylhydroxytoluene solution (1%) and evaporated under vacuum. The residue was dissolved in 100 µl of 25 mM ammonium acetate/acetonitrile (45%/55%), and 10 µl was injected into HPLC-MS (High Performance Liquid Chromatography-Mass Spectrum) system for rifampicin quantification. HPLC-MS system consisted of Agilent 1200 HPLC system (Agilent Technologies, Santa Clara, CA) coupled with QTRAP 6500 mass spectrometer (Sciex, Framingham, MA) using 25mM ammonium acetate/acetonitrile (45/55%) as the mobile phase and the reversed-phase column SB-C18 (2.1 mm×50 mm, particle size 1.8 μm, Agilent Technologies). Standard curve for rifampicin was constructed by making standard rifampicin solution and the intracellular rifampicin concentration was calculated from the standard curve to calculate the amount of rifampicin (ng) taken up per 10^6^ cells.

### Effects of Rif@IONPs-PAA-PEG-MAN nanodecoy on extracellualr Mtb growth

4 × 10^5^ colony-forming units (CFU) of BCG or H37Rv suspension in 7H9 medium was added into a 2 mL tube with IONPs-PAA-PEG-MAN, Rif@IONPs-PAA-PEG-MAN or rifampicin for 72 h treatment under slowly rotation. After that, the BCG or H37Rv suspension were diluted and plated on Middlebrook 7H11 plates. CFU counts on plates were measured at weeks 3–4 after the culture in incubator.

### Effects of Rif@IONPs-PAA-PEG-MAN nanodecoy on intracellualr Mtb growth

THP-1 cells were seeded at a density of 1 × 10^6^ into 12 well plates with 100 nM PMA stimulation for 24 h. Then, cells were infected with BCG using a MOI of 1 for 24 h or infected with H37Rv using a MOI of 1 for 4 h. After washed with PBS, IONPs-PAA-PEG- MAN, Rif@IONPs-PAA-PEG-MAN or rifampicin were added into the cells for 72 h incubation. After that, 0.03% SDS solution was used to lyse the cells for 15 min, and the cell lysis were plated onto Middlebrook 7H11 plates. CFU counts on plates were measured at weeks 3–4 after the culture in incubator. Freshly prepared PBMC (Peripheral Blood Mononuclear Cell) from healthy rhesus macaques were seeded into 24 well plate with a density of 1 × 10^7^/well for overnight incubation. After that, suspending cells were washed out using PBS, and the monocytes attached onto the substrate were digested by PBS containing 2 mM EDTA for 15 min. The collected monocytes were seeded into 96 well plate with a density of 1–2 × 10^4^/well for overnight incubation. Then, the monocytes were infected with H37Rv using a MOI of 1 for 4 h. After washed with PBS, IONPs-PAA-PEG-MAN, Rif@IONPs-PAA-PEG-MAN or rifampicin were added into the cells for 72 h incubation. After that, 0.03% SDS was used to lyse the monocytes for 15 min, and the cell lysis were plated onto Middlebrook 7H11 plates. CFU counts on plates were measured at weeks 3–4 after the culture in an incubator.

### In vivo **anti-TB effects of Rif@IONPs-PAA-PEG-MAN nanodecoy on Mtb infected mice**

Animal studies were approved by Institutional Animal Ethics Committee of Guangdong Medical University with approval number of GDY2202716, and studies were performed following the approved guidelines and the ethics of Institutional Animal Ethics Committee. BALB/c nude mice of 6 weeks old were intravenously injected with GFP-BCG (Green Fluorescent Protein-Bacillus Calmette Guerin) or DI@IONPs-PAA-PEG-MAN. IVIS (In Vivo Imaging System, PerkinElmer, USA) was used to monitor the distribution of GFP-BCG or DI@IONPs-PAA-PEG-MAN in mice in different time points. And after 72 h of injection, the mice was scarified, and the tissues were also imaged by the IVIS system to evaluate the distribution of GFP-BCG or DI@IONPs-PAA-PEG-MAN in mice. A systemic Mtb infection mice model was established to test the in vivo anti-TB effects of Rif@IONPs-PAA-PEG-MAN by attenuated Mtb strain H37Ra infection using a similar method previously reported [42]. 8 week-old female BALB/c mice were used for Mtb infection after 2 weeks of accommodation in the labs, approximately 1 × 10^7^ CFU of H37Ra resuspended in 200 µl saline were intravenously injected at the lateral tail vein of each mouse for infection. After 6 days of infection, mice were distributed into 4 groups for intravenous drug administration: (1) Control group (200 µl saline); (2) IONPs-PAA-PEG-MAN group (200 µl, 5 mg/kg); (3) Rif@IONPs-PAA-PEG-MAN group (200 µl, 5 mg/kg); (4) rifampicin group (200 µl, 50 µg/kg). 5 total drug administrations were performed in one month with a frequency of one administration/6 days. After 6 days of the last drug administration, mice were scarified and their blood and organs were harvested. The homogenized lysis of lungs were subsequently spread on 7H11 agar plates for CFU counting of H37Ra. Lungs, spleens, hearts, livers and kidneys were fixed and sliced for H&E-staining and microscope imaging to understand the tissue structures. Two important hepatic indicators (i.e., aspartate aminotransferase [AST] and alanine aminotransferase [ALT]) and two renal indicators (i.e., blood urea nitrogen [BUN] and creatinine [CRE]) were analyzed to estimate the potential systemic toxicity of drug treatment.

### Statistical analysis

All experiments were carried out at least in triplicate and results were expressed as mean ± standard error of mean. When there were more than two groups in the obtained data for comparative analysis, statistical analysis was performed using ANOVA-Tukey analysis (correct for multiple comparisons using statistical hypothesis testing). And when there were just two groups in the obtained data for comparative analysis, statistical analysis was performed using t-test analysis. p < 0.05 regarded as statistically significant in the comparative analysis of data.

## Results and discussion

### Preparation and characterization of Rif@IONPs-PAA-PEG-MAN nanodecoy

**Fig. 1:**
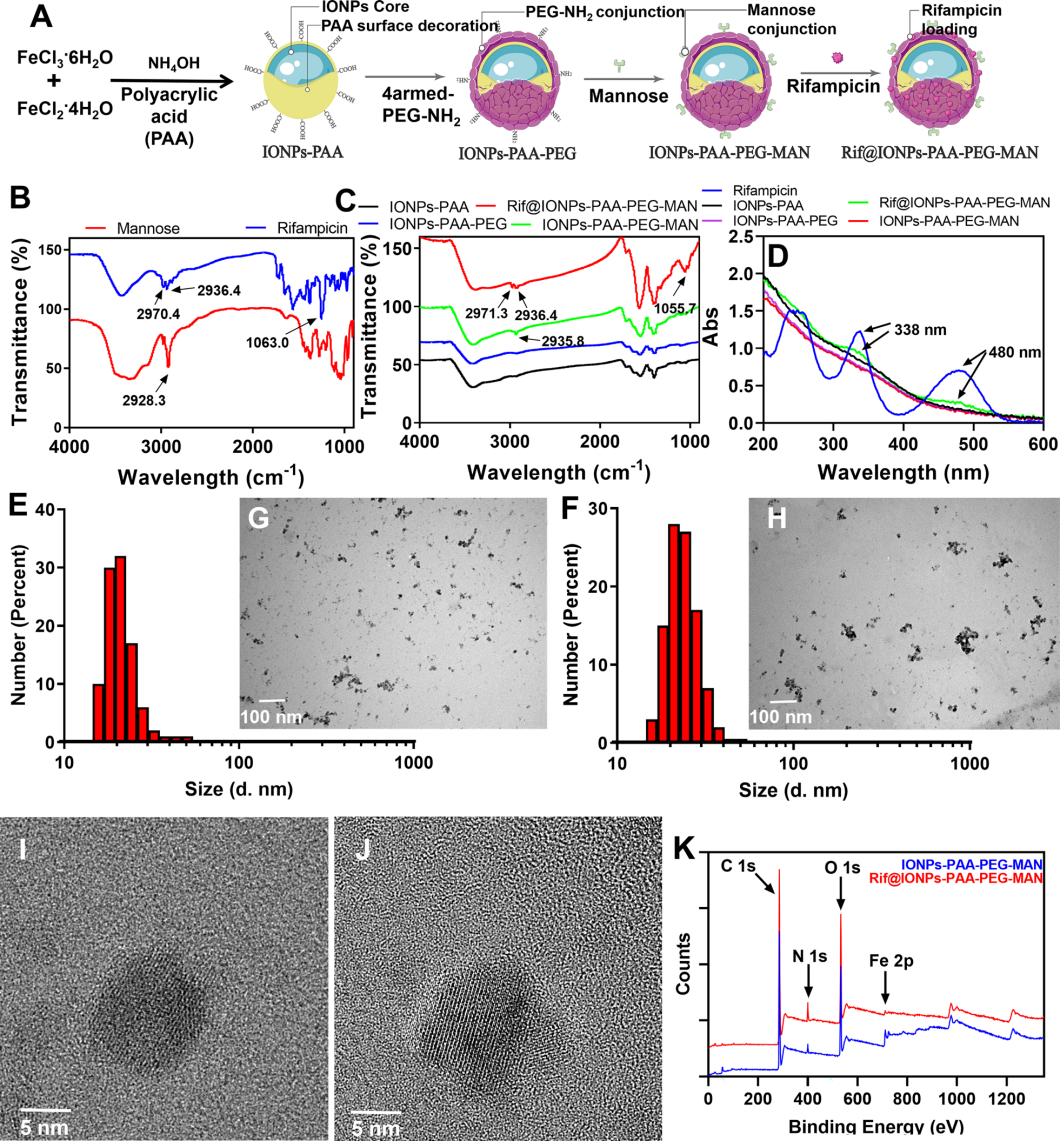
Preparation and characterization of IONPs-PAA-PEG-MAN and Rif@IONPs- PAA-PEG- MAN. **A** Schemes for the preparation of IONPs-PAA-PEG-MAN and Rif@IONPs-PAA-PEG-MAN. **B** FTIR analysis of mannose and rifampicin. **C** FTIR analysis of IONPs-PAA, IONPs-PAA-PEG, IONPs-PAA-PEG-MAN and Rif@IONPs-PAA- PEG-MAN. **D** UV–Vis analysis of rifampicin, IONPs-PAA, IONPs-PAA-PEG, IONPs- PAA-PEG-MAN and Rif@IONPs-PAA-PEG-MAN. Size distribution of (**E**) IONPs- PAA-PEG-MAN and **F** Rif@IONPs-PAA-PEG-MAN analyzed by DLS. TEM image of (**G**) IONPs-PAA-PEG-MAN and **H** Rif@IONPs-PAA-PEG-MAN, scale bar: 100 nm. High-resolution TEM image of (**I**) IONPs-PAA-PEG-MAN and (**J**) Rif@IONPs-PAA-PEG- MAN, scale bar: 5 nm. **K** X-ray photoelectron spectroscopy (XPS) analysis of IONPs-PAA- PEG-MAN and Rif@IONPs-PAA-PEG-MAN

Here, a versatile method was applied to synthesize IONPs-PAA-PEG-MAN and rifampicin incorporated Rif@IONPs-PAA-PEG-MAN (Fig. 1A), which was expected to act as macrophage-targeted rifampicin delivery system and innate immunity manipulation nanodecoy. Polyacrylic acid (PAA) stabilized iron oxide nanoparticles (IONPs) were prepared as previously reported with the decoration of PAA polymer molecules on the surface [38], which provided plenty of carboxyl groups for the surface conjugation of 4-armed PEG-NH_2_ to form IONPs-PAA-PEG with free amino groups at the PEG end. The acidic environment resulted in the ring opening of the mannose molecules, causing the aldehyde group to react with the free amine group from 4-armed PEG-NH_2_ and then forming IONPs-PAA-PEG-MAN. After these processes, the hydrophobic antibiotics rifampicin could be further encapsulated into the outer PAA and PEG polymer warehouse of the nanoparticles by multiple hydrogen bonds to yield a novel anti-TB therapeutic drug loaded nanodecoy, naming Rif@IONPs-PAA-PEG-MAN.

By FTIR spectroscopy analysis, mannose showed specific FTIR absorption peaks at 2928.3 cm-1 for -CH- groups, while IONPs-PAA-PEG-MAN and Rif@IONPs-PAA- PEG-MAN showed similar absorption peaks at 2935.8 cm-1 and 2936.4 cm-1, respectively (Fig. 1B, C), demonstrating the successful mannosylation of IONPs. Rifampicin showed specific FTIR absorption peaks at 1063.0 cm-1 for -C-O-C- group, and Rif@IONPs-PAA-PEG-MAN showed similar absorption peaks at 1055.7 cm-1 for the -C-O-C- group from rifampicin (Fig. 1B, C), confirming the successful encapsulation of rifampicin into IONPs-PAA-PEG-MAN. UV-Vis spectroscopy analysis of Rif@IONPs-PAA- PEG-MAN also indicated the specific absorption peak of rifampicin at 338 and 480 nm (Fig. 1D), which further confirmed the successful encapsulation of rifampicin in Rif@IONPs-PAA-PEG-MAN.

The size of nanoparticles in water solution was further analyzed by DLS, which showed average diameters of 20 and 22 nm for IONPs-PAA-PEG-MAN and Rif@IONPs-PAA-PEG-MAN, respectively (Fig. 1E, F). However, further TEM analysis (Fig. 1G, H) and high resolution TEM analysis (Fig. 1I, J) revealed an average iron oxide core of 10 nm for both IONPs-PAA-PEG-MAN and Rif@IONPs-PAA-PEG-MAN. The larger hydrous diameter for IONPs-PAA-PEG-MAN and Rif@IONPs-PAA-PEG-MAN than that of TEM imaging results suggested the formation of a thick PAA and PEG polymeric coating (about 5 to 6 nm in radius) around the iron oxide nanoparticle core that can be detected by DLS but can’t be clearly observed by TEM imaging due to their low electron density. And this polymeric coating around the iron oxide nanoparticle core thus provided an ideal warehouse for the encapsulation of hydrophobic guest molecules (such as rifampicin) for further drug loading and delivery.

To clearly explore the composition of Rif@IONPs-PAA-PEG-MAN nanodecoy, we also applied elemental mapping and TEM based EDS (Energy Dispersive Spectrometer) analysis of the obtained nanoparticles (Additional file 1: Fig. S1), which confirmed the presence of iron element and oxygen element in IONPs-PAA-PEG-MAN and Rif@IONPs-PAA-PEG-MAN. The XRD analysis of IONPs-PAA-PEG-MAN and Rif@IONPs-PAA-PEG-MAN both indicated characteristic peaks of Fe_3_O_4_ magnetite around 30, 35.5, 43, 57, and 63 degrees in 2-Theta scale (Additional file 1: Fig. S2), which were similar with some reported Fe_3_O_4_ magnetite nanoparticles [43, 44]. However, the loading of rifampicin into Rif@IONPs-PAA-PEG-MAN changed the characteristic peaks of Fe_3_O_4_ magnetite at 43 degree to 42 degree (Additional file 1: Fig. S2). Moreover, Rif@IONPs-PAA-PEG-MAN showed higher intensity and narrower peak width than that of IONPs-PAA-PEG-MAN (Additional file 1: Fig. S2), which indicated that rifampicin loading inside Rif@IONPs-PAA-PEG-MAN would result in larger particle size than IONPs-PAA-PEG-MAN.

**Fig. 2:**
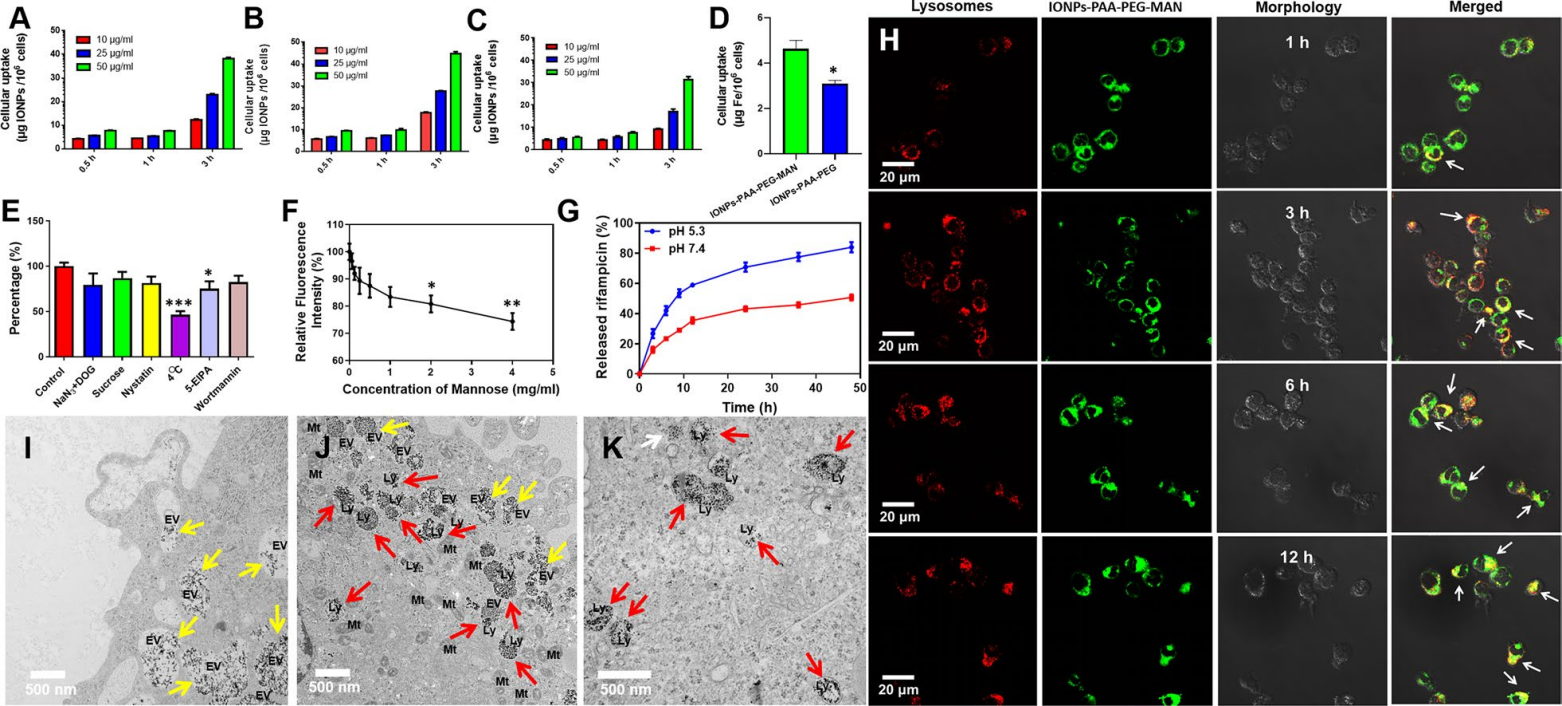
Cellular uptake and intracellular localization of IONPs-PAA-PEG-MAN in macrophages. Time-dependent and dose-dependent cellular uptake of (**A**) C6@ IONPs-PAA-PEG and (**B**) C6@IONPs-PAA-PEG-MAN in THP-1 cells, n = 3. **C** Time-dependent and dose-dependent cellular uptake of C6@IONPs-PAA-PEG-MAN in HLMVEC cells, n = 3. **D** Cellular uptake of IONPs-PAA-PEG-MAN and IONPs- PAA-PEG in THP-1 cells after 1 h treatment by analyzing Fe (iron element) concentration, n = 3, t-test analysis was applied for the comparative analysis of data, *p < 0.05. **E** Cellular uptake of C6@IONPs-PAA-PEG-MAN by THP-1 cells under different endocytosis inhibition conditions, control group is treated without any inhibition conditions for C6@IONPs-PAA-PEG-MAN uptake analysis, n = 3, ANOVA-Tukey analysis was applied for the comparative analysis of data, *p < 0.05, ***p < 0.001. **F** Intracellular uptake of C6@IONPs-PAA-PEG-MAN by THP-1 cells with free mannose competition, control group is treated without mannose for C6@IONPs-PAA-PEG-MAN uptake analysis, n = 3, ANOVA-Tukey analysis was applied for the comparative analysis of data, *p < 0.05, **p < 0.01. **G** In vitro drug release of Rif@IONPs-PAA-PEG-MAN under different pH environments, n = 3. **H** Fluorescence imaging for intracellular localization of C6@IONPs-PAA-PEG-MAN with lysosomes in THP-1 cells, white arrows indicate the co-localization of IONPs-PAA-PEG-MAN with lysosomes, scale bar: 20 μm. **I–K** Representative TEM images of THP-1 cells after incubation with IONPs-PAA-PEG- MAN, yellow arrows indicate the IONPs-PAA-PEG-MAN in endocytosis vesicles (EV) at the early stage of cell uptake, red arrows indicate the IONPs-PAA- PEG-MAN in lysosomes and white arrows indicate the IONPs-PAA-PEG-MAN in the cytoplasm, scale bar: 500 nm. For TEM imaging, THP-1 cells were seeded at a density of 1 × 10^6^ into 6 plates with 100 nM PMA stimulation for 24 h. Cells were treated with IONPs-PAA-PEG-MAN for 12 h, and then collected, washed with PBS and fixed by 2.5% glutaraldehyde and 2% paraformaldehyde for 48 h at 4 ℃. The fixed cell samples were washed with PBS and then further fixed with 0.1% osmic acid for 2 h. After washed with PBS, the samples were dehydrated with sequential treatment of 50%, 70%, 85%, 90%, and 100% ethanol, respectively. Then, the samples were embedded in resin, cut into ultrathin slices, stained with 2% uranyl acetate and 0.2% lead citrate before TEM observation

Moreover, we also applied XPS analysis of the proposed nanoparticles, which indicated increased N 1s signal in Rif@IONPs-PAA-PEG-MAN compared with that of IONPs-PAA- PEG-MAN (Fig. 1K), demonstrating the successful loading of rifampicin into Rif@IONPs- PAA-PEG-MAN. The detailed analysis of Fe 2p about these nanoparticles indicated similar characteristic peak of Fe 2p (Additional file 1: Fig. S3) with some reported Fe_3_O_4_ magnetite nanoparticles [45, 46], which indicated the presence of Fe^3+^ and Fe^2+^ in form of Fe_3_O_4_ magnetite in Rif@IONPs-PAA-PEG-MAN. And by analysis of rifampicin contents in Rif@IONPs-PAA-PEG-MAN, we found that there were 9.89 µg of rifampicin in 1 mg of nanoparticles (Data not shown), which indicated a loading efficiency of 1% and an encapsulation efficiency of 4% for rifampicin in Rif@IONPs-PAA-PEG-MAN.

**Fig. 3:**
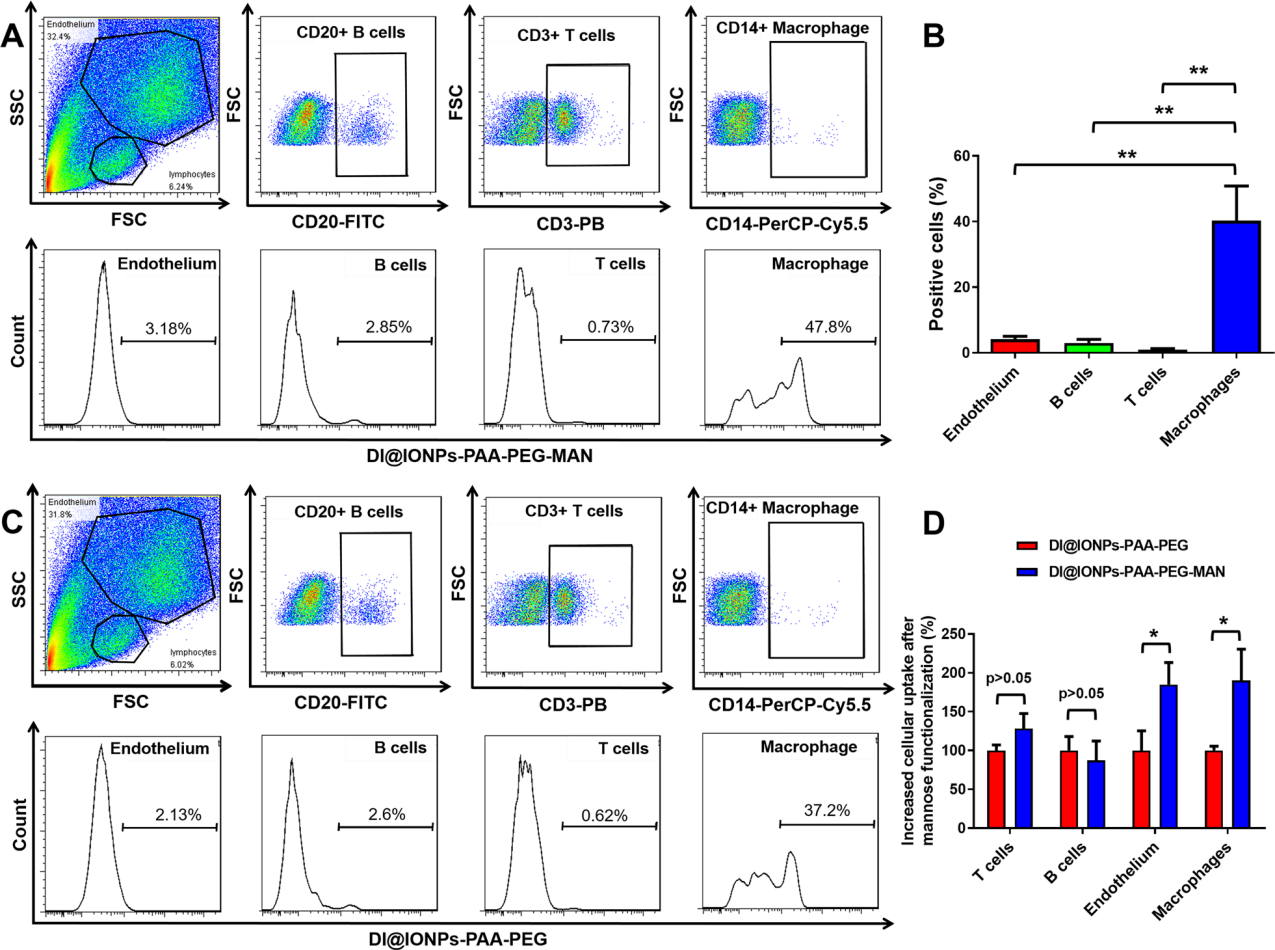
Selective cellular uptake of IONPs-PAA-PEG-MAN by primary macrophages from intraepithelial lymphocytes of Mtb infected rhesus macaques. **A** Cellular uptake of DI@IONPs-PAA-PEG-MAN by endothelium, T cells, B cells and macrophages from the intraepithelial lymphocytes of rhesus macaqus after 3 h treatment. **B** Statistical analysis for cellular uptake of DI@IONPs-PAA-PEG-MAN by endothelium, T cells, B cells and macrophages from the intraepithelial lymphocytes of rhesus macaques after 3 h treatment, n = 3, ANOVA-Tukey analysis was applied for the comparative analysis of data, **p < 0.01. **C** Cellular uptake of DI@IONPs-PAA-PEG-MAN by endothelium, T cells, B cells and macrophages from the intraepithelial lymphocytes of rhesus macaqus after 3 h treatment. **D** Statistical analysis for the cellular uptake of DI@IONPs-PAA-PEG-MAN and DI@IONPs- PAA-PEG in endothelium, T cells, B cells and macrophages from the intraepithelial lymphocytes of rhesus macaqus after 3 h treatment, the positive cell percentages of DI@IONPs-PAA-PEG treated cells were set as the criteria to determine the increased cellular uptake of DI@IONPs-PAA-PEG-MAN with mannose modification, n = 3, multiple t-test analysis was applied for the comparative analysis of data, *p < 0.05

Thus, based on these results, we proposed the structure of Rif@IONPs-PAA-PEG-MAN in water solution as shown in Fig. 1A. There are an iron oxide nanoparticle core (Fe_3_O_4_ magnetite) with lots of PAA polymer molecules decorated on the surface. Further PEG and mannose modification thus allows the formation of a thick polymeric coating on the surface of iron oxide nanoparticle core. In water solution, the PAA and PEG polymeric coating could form a hydrous warehouse for rifampicin loading and further drug delivery application.

### In vitro **cellular uptake, intracellular localization and drug release behaviors of Rif@IONPs-PAA-PEG-MAN nanodecoy**

We found that the proposed IONPs-PAA-PEG-MAN exhibited low or no cytotoxicity in several different kinds of cells, including THP-1 cells, RAW264.7 macrophages, HLMVEC and A549 cells (Additional file 1: Fig. S4), demonstrating their low cytotoxicity and high biocompatibility for biomedical uses. We then analyzed the cellular uptake of the nanodecoys in cells using a similar method as previously described [47–49]. Cellular uptake analysis of coumarin-6-loaded C6@IONPs-PAA-PEG (Fig. 2A) and C6@IONPs-PAA-PEG-MAN (Fig. 2B) in THP-1 macrophages both showed time and dose dependent increases. The significant higher cellular uptake of IONPs-PAA-PEG-MAN than IONPs-PAA-PEG (Additional file 1: Fig. S5A–C) in THP-1 macrophages were consistent with our hypothesis for the enhanced macrophage uptake of IONPs after mannose conjugation. The much higher cellular uptake of C6@IONPs-PAA-PEG-MAN in THP-1 than that of HLMVEC cells (Fig. 2B, C and Additional file 1: Fig. S5D–F) further implicated the in vitro selectivity of IONPs-PAA-PEG-MAN against macrophages. To further confirm the enhanced cellular uptake of IONPs-PAA-PEG-MAN in THP-1 macrophages beyond IONPs-PAA-PEG, we applied ICP-AES analysis of intracellular Fe (iron element) concentration in THP-1 cells using a similar method as previously described for metal ion analysis [40]. The obtained results indicated that there were almost 4.3 μg of iron in 10^6^ THP-1 cells after IONPs-PAA-PEG-MAN, which were significantly higher than that of IONPs-PAA-PEG (Fig. 2D). These results collectively suggested that mannose modification could significantly enhance the cellular uptake of the nanodecoy system in macrophages.

To dissect the mechanisms for macrophage-targeted cellular uptake of IONPs-PAA-PEG-MAN, THP-1 cells were pre-treated with different uptake inhibition conditions before the addition of C6@IONPs-PAA-PEG-MAN. Firstly, treatments of NaN_3_ in combination with 2-deoxy-Dglucose (DOG), or low temperature (4℃), strongly inhibited the C6@IONPs- PAA-PEG-MAN internalization by 79.3% and 46.5% compared to control (Fig. 2E), The specific inhibitor of clathrin-mediated endocytosis, sucrose, decreased the endocytosis/uptake of C6@IONPs-PAA-PEG-MAN in THP-1 cells by 87.0% compared to the control (Fig. 2E), suggesting that clathrin-mediated endocytosis partially contributed to the macrophage-targeted cellular uptake of IONPs-PAA-PEG-MAN. The inhibitor of lipid raft-dependent endocytosis, nystatin, reduced the C6@IONPs-PAA-PEG-MAN uptake by 84% (Fig. 2E), which indicated that lipid raft-mediated endocytosis was also involved in the macrophage-targeted uptake of IONPs-PAA-PEG-MAN by macrophages. 5-(N-Ethyl-N- isopropyl) amiloride (5-EIPA) or wortmannin, the inhibitor for macropinocytosis/phagocytosis, reduced the cellular uptake to 75.0% and 82.3% compared to control (Fig. 2E). Comparative analysis indicated that only low temperature (4℃) and 5-EIPA treatment significantly reduced the cellular uptake of IONPs-PAA-PEG-MAN, which demonstrated that IONPs-PAA-PEG-MAN was mainly transported into macrophages by means of energy-dependent endocytosis and macropinocytosis/phagocytosis.

To further explore the contribution of mannose receptor in the cellular uptake of IONPs-PAA-PEG-MAN in macrophages, THP-1 cells were pre-treated with excess amount of free mannose before the addition of C6@IONPs-PAA-PEG-MAN. As shown in Fig. 2F, free mannose significantly inhibited the macrophage-targeted uptake of C6@IONPs-PAA-PEG-MAN in a dose-dependent manner. 2 mg/ml and 4 mg/ml mannose pre-treatment significantly reduced the cellular uptake of IONPs-PAA-PEG-MAN, with nearly 26% of cellular uptake inhibition upon 4 mg/ml mannose pre-treatment. These results suggested that mannose-mannose receptor interaction impacted the selective cellular uptake of IONPs-PAA-PEG-MAN in macrophages, which was consistent with the appealing hypothesis that mannose surface decoration could increase the macrophage targeting effects of IONPs-PAA-PEG-MAN.

It has been well described that controlled drug release is one of the superior properties of nano-structured delivery system beyond the free drugs, which can lead to selective drug release into the precise site of the diseased cells or into the precise organelles of targeted cells, resulting in better drug efficiency and lower drug toxicity [50]. To explore the drug release behaviors of Rif@IONPs-PAA-PEG-MAN after cellular uptake, PBS solutions at pH 7.4 was used to simulate the blood or cytoplasm environments and PBS solutions at pH 5.3 was used to simulate the acidic lysosomal environments in vivo. As indicated in Fig. 2G, the cumulative release amount of rifampicin from the nanosystem at pH 5.3 was nearly 26.78 ± 1.74% for 3 h and nearly 83.98 ± 1.94% for 48 h, whereas the release rate at pH 7.4 was 16.02 ± 1.19% for 3 h and finally reached 50.73 ± 1.13% for 48 h. The significant higher rifampicin release at pH 5.3 indicated the pH sensitive and controlled release of rifampicin from Rif@IONPs-PAA-PEG- MAN under acidic conditions, which also implied that the acidic lysosomal environment might accelerate the drug release of Rif@IONPs-PAA-PEG-MAN. The increased rifampicin release from Rif@IONPs-PAA-PEG-MAN nanodecoy in acidic condition may mainly be attributed to PEG polymer erosion and degradation [51] and reduced electrostatic interaction between PAA [52] under acidic conditions. Moreover, the increased rifampicin release from Rif@IONPs-PAA-PEG-MAN nanodecoy in acidic condition may also be partially attributed to the disintegration of iron oxide core in the acidic lysosome environment. As iron oxide core is closely stabilized by PAA (Polyacrylic acid), the disintegration of iron oxide core would also induce significant structural changes of PAA polymer core and the PEG polymer linked with PAA polymer, which may induce increased drug release of encapsulated rifampicin in the polymers.

The fates of nanomaterial-based drug delivery system in cells, such as toxicity, drug release behavior and degradation, are closely related to their intracellular localization, especially the direct exposure against some organelles [53]. Lysosome, the most important organelle for cellular degradation functions, has been found to play critical roles in the intracellular transport and degradation of nanomaterials [12, 54]. Here, our fluorescence imaging demonstrated that C6@IONPs-PAA-PEG-MAN moved cross the membrane, entered into the cytoplasm and partially accumulated into the lysosomes at 1 h (Fig. 2H). After continuous incubation for 3 h, 6 and 12 h, more and more C6@IONPs-PAA-PEG-MAN accumulated into the lysosomes with very bright, strong yellow fluorescence (Indicated by white arrows, Fig. 2H), suggesting that lysosome was the main target organelle of IONPs-PAA-PEG-MAN in macrophages. Using the TEM imaging (Fig. 2I–K), we found that most of IONPs-PAA-PEG-MAN initiated the endocytosis processes at the cell membrane, entered macrophages through the endocytosis vesicles (Indicated by yellow arrow), and then predominantly accumulated in the lysosomes (Indicated by red arrow). These TEM observations further suggested that lysosome was the main target organelle of IONPs-PAA- PEG-MAN in macrophages. And considering the facts that Rif@IONPs-PAA-PEG-MAN could rapidly release rifampicin under acidic conditions, the lysosomal acidic environment would lead to drastic rifampicin release after the entry of Rif@IONPs-PAA-PEG-MAN into lysosomes.

### Ex vivo **cellular uptake of IONPs-PAA-PEG-MAN nanodecoy**

Our above results with in vitro settings have proved that IONPs-PAA-PEG-MAN based drug delivery system possess selective targeting effects in macrophage cell lines (Fig. 2), which highlight their potential uses for host cell directed therapies against TB. Then, we sought to explore the potential ex vivo targeting effects of IONPs-PAA-PEG-MAN in primary macrophages from Mtb infected animal model, which was critical for their further anti-TB applications. To this end, we investigated the ex vivo uptake of DI@IONPs-PAA-PEG-MAN in intraepithelial lymphocytes isolated from the small intestine of Mtb infected rhesus macaques. As shown in Fig. 3A, B, the ex vivo results indicated much and significantly higher cellular uptake of DI@IONPs-PAA-PEG-MAN by intestinal macrophages than that of T cells, B cells and endothelium after 3 h treatments, which therefore demonstrated the selective ex vivo targeting effects of IONPs-PAA-PEG-MAN in primary macrophages from Mtb infected animal model.

Furthermore, we also compared the ex vivo cellular uptake of IONPs-PAA-PEG-MAN and IONPs-PAA-PEG in these intraepithelial lymphocytes isolated from Mtb infected rhesus macaques. As shown in Fig. 3, we found that mannose surface modification led to the much and significantly higher cellular uptake of DI@IONPs-PAA-PEG-MAN in macrophages and endothelium than that of DI@IONPs-PAA-PEG, where the increased cellular uptake in endothelium might be attributed to the non-negligible mannose receptor expression in endothelium. However, the totally cellular uptake of DI@IONPs-PAA-PEG-MAN by macrophages was still much higher than that in endothelium (Fig. 3). These results collectively suggested the macrophage targeting effects of IONPs-PAA-PEG-MAN, which therefore showed their potentials to be served as a kind of selective macrophage-targeting system for TB therapy relevant to the in vivo settings.

### Intracellular localization of IONPs-PAA-PEG-MAN Nanodecoy and Mtb in macrophages

Macrophages function as critical mediators of innate immunity of antimicrobial response by a plethora of phagosome engulfing of microorganisms, phagosome-lysosome fusion, killing and digestion processes. However, one of the most important pathogenesis hallmarks for TB is the immune escape of Mtb from lysosomal destruction in macrophages by inhibiting their fusion into lysosomes, which further subverts the host immunity mechanisms of intracellular bacteria killing and antigen presentation [2–4]. As most of the intracellular stubborn Mtb escaped from the lysosomes are hiding in the phagosomes for survival, it’s necessary to develop new strategies to effectively kill the Mtb hided in phagosomes.

Since we already demonstrated that lysosome was the main target organelle of IONPs-PAA-PEG-MAN in macrophages, the high iron contents of IONPs-PAA-PEG-MAN in lysosomes thus would be a tempting granary for Mtb, highlighting IONPs-PAA- PEG-MAN as a kind of novel nanodecoy to abduct the Mtb in phagosomes. The use of IONPs as “Trojan horses” for antibiotic delivery has been explored previously to shown enhanced Mtb killing effects beyond the free antibiotic-isoniazide [23], but the detailed roles/mechanisms of the lysosomes and anti-Mtb immunological responses upon IONPs treatment were rarely explored. Moreover, their previously published IONPs system didn’t show selective macrophage targeting effects, which are very important properties to enhance the drug efficiency with reduced side effects and less potentials to develop drug-resistance. Here, as macrophage-targeted IONPs-PAA-PEG-MAN massively accumulated in lysosomes, we employed Rif@IONPs-PAA-PEG-MAN as the nanodecoy to enhance the anti-microbial innate immunity and deliver large amounts of antibiotics into the Mtb localized intracellular sites for more effective killing of intracellular stubborn Mtb.

Firstly, we examined the localization patterns of GFP-BCG (BCG tagged with green fluorescent proteins), DI@IONPs-PAA-PEG-MAN and lysosomes by fluorescence imaging, which proved that after the phagolysosome fusion, GFP-BCG could efficiently co-localize with DI@IONPs-PAA-PEG-MAN in lysosomes (Additional file 1: Fig. S6). These results demonstrated the potential ability of Rif@IONPs-PAA-PEG-MAN as a kind of novel naonodecoy to co-localize with Mtb, which provided the convenience for the rapid release of encapsulated drugs for direct killings of Mtb.

**Fig. 4:**
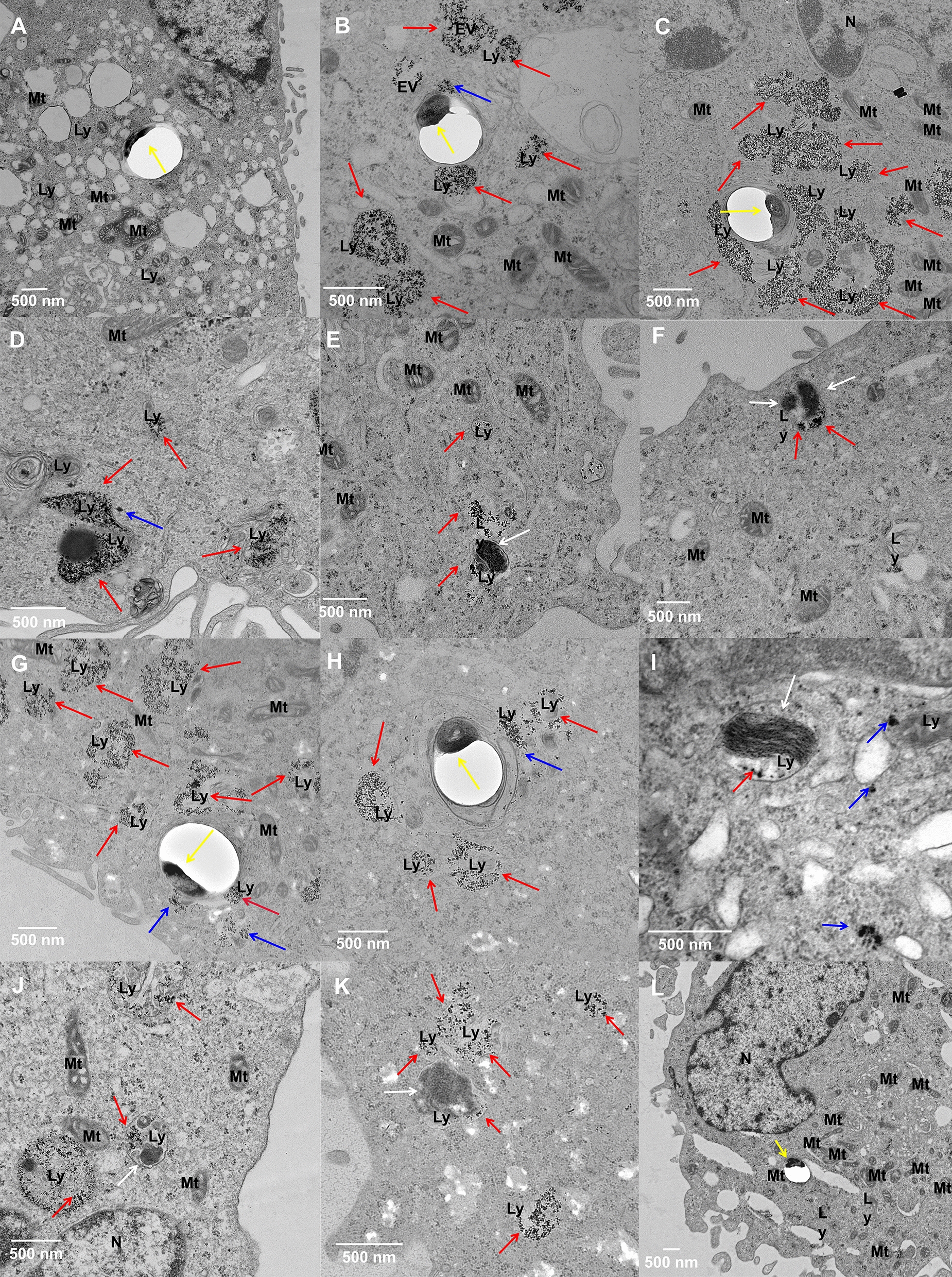
TEM imaging of IONPs-PAA-PEG-MAN and Rif@IONPs-PAA-PEG-MAN promoted M.tb-nanodecoy co-localization for intracellular Mtb killings in THP-1 macrophages. **A** Control THP-1 macrophages infected with H37Rv, H37Rv in phagosome was indicated by yellow arrow. **B**, **C** H37Rv infected THP-1 macrophages after IONPs-PAA-PEG-MAN treatment, H37Rv in phagosomes (indicated by yellow arrow) were surrounded by IONPs-PAA-PEG-MAN in lysosomes (indicated by red arrow) and IONPs-PAA-PEG-MAN in endosomes (indicated by blue arrow). **D–F** H37Rv infected THP-1 macrophages after IONPs-PAA-PEG-MAN treatment, some H37Rv (indicated by white arrow) were fused into lysosomes or located in lysosomes with IONPs-PAA-PEG-MAN (indicated by red arrow) co-localized inside. **G**, **H** H37Rv infected THP-1 macrophages after Rif@IONPs-PAA- PEG-MAN treatment, H37Rv in phagosomes (indicated by yellow arrow) were surrounded by Rif@IONPs-PAA-PEG-MAN in lysosomes (indicated by red arrow) and Rif@IONPs- PAA-PEG-MAN in cytoplasm (indicated by blue arrow). **I**–**K** H37Rv infected THP-1 macrophages after Rif@IONPs-PAA-PEG-MAN treatment, some H37Rv (indicated by white arrow) were fused into or located in lysosomes with Rif@IONPs-PAA-PEG-MAN (indicated by red arrow) co-localized inside. H37Rv in lysosomes from (**J**–**K**) were partially destroyed by Rif@IONPs-PAA-PEG-MAN to show very incompact and penetrable cross section morphology. **L** H37Rv infected THP-1 macrophages after rifampicin treatment, H37Rv in phagosome was indicated by yellow arrow. For TEM imaging, THP-1 cells were seeded at a density of 1 × 10^6^ into 6 plates with 100 nM PMA stimulation for 24 h, and then infected with H37Rv (4 h infection) using MOI = 1. The infected cells were treated with IONPs-PAA-PEG- MAN or Rif@IONPs-PAA-PEG-MAN for 72 h, and then collected, washed with PBS and fixed by 2.5% glutaraldehyde and 2% paraformaldehyde for 48 h at 4 ℃. The fixed cell samples were washed with PBS and then further fixed with 0.1% osmic acid for 2 h. After washed with PBS, the samples were dehydrated with sequential treatment of 50%, 70%, 85%, 90%, and 100% ethanol, respectively. Then, the samples were embedded in resin, cut into ultrathin slices, stained with 2% uranyl acetate and 0.2% lead citrate before TEM observation

We then employed high resolution TEM imaging to explore precise mechanistic details in H37Rv infected THP-1 macrophages with IONPs-PAA-PEG-MAN or Rif@IONPs-PAA- PEG-MAN treatment. Control THP-1 cells infected with H37Rv alone always showed representative H37Rv bacilli outside lysosomes (Fig. 4A). However, with IONPs-PAA-PEG- MAN (Fig. 4B, C and Additional file 1: Fig. S7) or Rif@IONPs-PAA-PEG-MAN (Fig. 4G, H and Additional file 1: Fig. S7) treatment, the IONPs-PAA-PEG-MAN (Fig. 4D–F) or Rif@IONPs-PAA-PEG-MAN (Fig. 4I–K) accumulated lysosomes were found to localize very close to the H37Rv contained phagosomes, and some of the Mtb even started to fuse into the nanodecoy contained lysosomes, which provided the convenience for direct Mtb killings by rifampicin released from the nanodecoy. It was also noteworthy that most H37Rv bacilli co-localizing with Rif@IONPs-PAA-PEG-MAN in lysosomes exhibited incompact and penetrable cross-section morphology (destruction-like structure), and consistently the internal H37Rv structure seemed to be destroyed into pieces, compared to other control groups (Fig. 4K and Additional file 1: Fig. S8). Thus, our proposed Rif@IONPs-PAA-PEG-MAN nanodecoy could partially promote lysosomal destruction of Mtb, which further enhanced the killing effects of Rif@IONPs-PAA-PEG-MAN released rifampicin against intracellular Mtb.

It has also been known that iron, as an essential element for Mtb metabolism, is always not freely available in the host, making Mtb to actively compete for this metal to maintain growth and establish an infection [18]. However, excess iron could also be extremely toxic to organisms [18–20]. Here, the proposed Rif@IONPs-PAA-PEG-MAN nanosystem provides excess iron that might abduct the Mtb to where the Rif@IONPs-PAA-PEG-MAN localized, which leads to the direct drug exposure and more effective drug killings. On the other hand, as excess iron could also be extremely toxic [18–20], the toxicity of excessive iron from the iron oxide nanoparticles might also contribute to the killing effects of Rif@IONPs-PAA- PEG-MAN. And additionally, IONPs-PAA could also act as a new class of efflux inhibitors to enhance the efficiency of anti-TB antibiotics for more effective Mtb killing [55], which might also contribute to the killing effects of Rif@IONPs-PAA-PEG-MAN. However, rifampicin treated THP-1 cells as control group didn’t induce such synergistic killing effects, and in this control setting, most H37Rv bacilli localized outside lysosomes and maintained their intact morphology (Fig. 4L). Therefore, the effects above appeared to act in concert to synergistically enhance killing effects of Rif@IONPs-PAA-PEG-MAN as novel nanodecoy on intracellular Mtb.

### Effects of Rif@IONPs-PAA-PEG-MAN nanodecoy on the polarization of Mtb infected macrophages

Macrophage functions are settled in response to micro-environmental signals to drive the polarization programs, whose extremes are simplified in the classical (M1) or alternative (M2) activation state. Functional skewing of monocyte/macrophage polarization occurs in physiological conditions (e.g., ontogenesis and pregnancy), as well as in pathology (allergic and chronic inflammation, tissue damage/repair, infection, and cancer) and is now considered as a key determinant of disease development and/or regression [56]. M1 type macrophages release high levels of pro-inflammatory cytokines that exhibit high anti-mycobacterial activity; on the contrary, M2 type macrophages produce inhibitory cytokines that are associated with weakening of the anti-bacterial and particularly anti-TB defense [57]. IONPs have been reported to be a kind of innate immune activation agents inducing pro-inflammatory polarization of macrophages [24, 25]. Thus, to determine whether IONPs-PAA-PEG-MAN could impact the polarization of Mtb infected macrophages, we tested the expression of M1 macrophage marker-CD80 and M2 macrophage marker-CD206 in Mtb infected THP-1 cells, respectively.

**Fig. 5:**
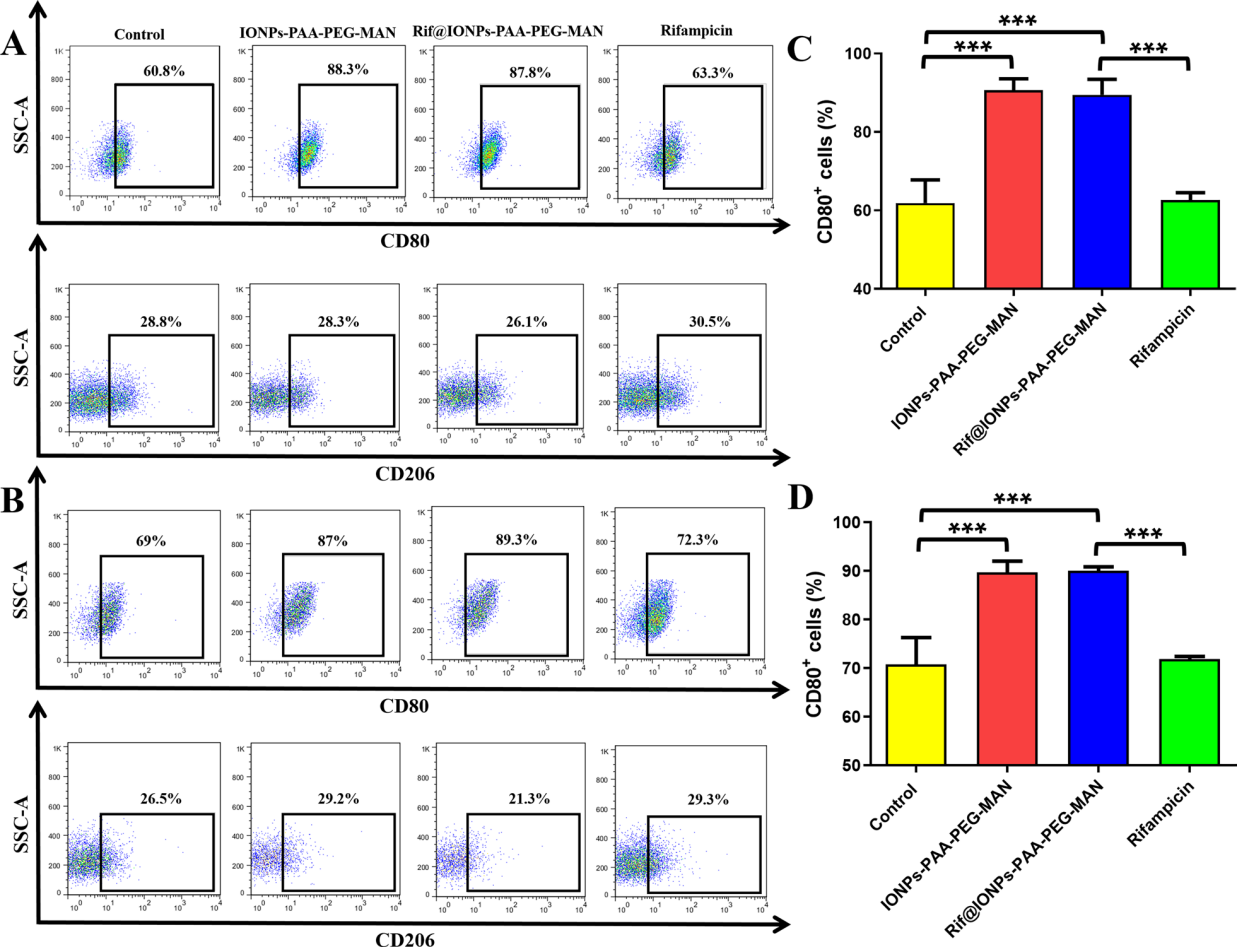
Effects of IONPs-PAA-PEG-MAN and Rif@IONPs-PAA-PEG-MAN on the polarization of M.tb-infected macrophages. **A** Flow cytometry analysis of CD80 positive cells and CD206 positive cells in BCG-infected THP-1 macrophages after IONPs-PAA-PEG-MAN, rifampicin and Rif@IONPs-PAA-PEG-MAN treatment, control group means BCG-infected THP-1 macrophages without drug treatment. **B** Flow cytometry analysis of CD80 positive cells and CD206 positive cells in H37Rv-infected THP-1 macrophages after IONPs-PAA-PEG-MAN, rifampicin and Rif@IONPs-PAA-PEG-MAN treatment, control group means H37Rv-infected THP-1 macrophages without drug treatment. Statistical analysis for the effects of IONPs-PAA-PEG-MAN, rifampicin and Rif@IONPs-PAA-PEG-MAN on the percentage of CD80 positive cells in (**C**) BCG-infected THP-1 macrophages and (**D**) H37Rv-infected THP-1 macrophages, control group means BCG or H37Rv-infected THP-1 macrophages without drug treatment, n = 3, ANOVA-Tukey analysis was applied for the comparative analysis of data, ***p < 0.001

As shown in Fig. 5A, C, IONPs-PAA-PEG-MAN could significantly increase the percentage of M1 type macrophages and decrease the percentage of M2 type macrophages at a dose-dependent fashion in BCG-infected THP-1 cells. Rif@IONPs-PAA-PEG-MAN could also significantly increase the percentage of M1 type macrophages and decrease the percentage of M2 type macrophages in BCG-infected THP-1 cells while rifampicin alone did not show similar effects (Fig. 5A, C). Similarly, in the setting of H37Rv infection, both IONPs-PAA-PEG-MAN and Rif@IONPs-PAA-PEG-MAN treatment could also increase the percentage of M1 type macrophages and decrease the percentage of M2 type macrophages at dose-dependent action in H37Rv-infected THP-1 macrophages (Fig. 5B, D). These results collectively suggested that IONPs-PAA-PEG-MAN and Rif@IONPs-PAA-PEG-MAN could significantly promote M1 pro-inflammatory/anti-microbial polarization of Mtb infected macrophages, which was closely associated with IONPs-PAA-PEG-MAN-enhanced innate anti-TB immunity against Mtb.

**Fig. 6:**
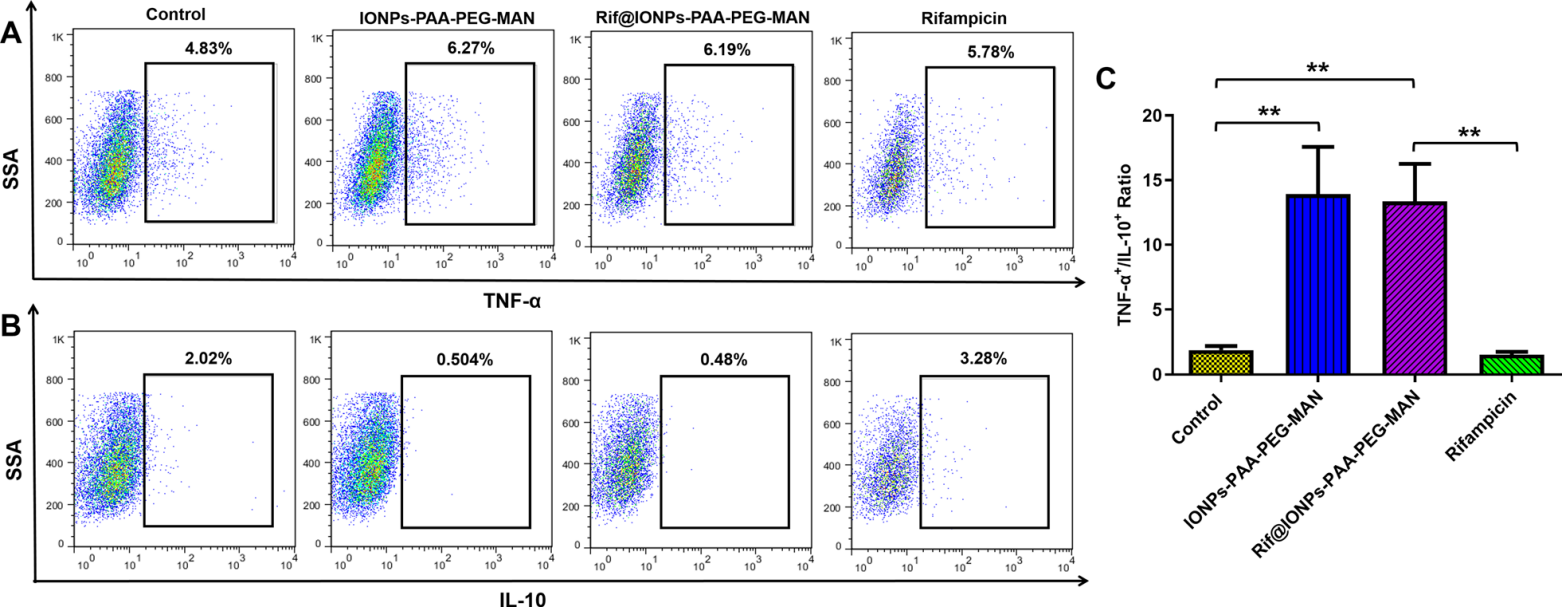
Effects of IONPs-PAA-PEG-MAN and Rif@IONPs-PAA-PEG-MAN on the expression of pro-inflammatory cytokine TNF-α and anti-inflammatory cytokine IL-10 in Mtb-infected macrophages. Expression of (**A**) TNF-α and **B** IL-10 in H37Rv-infected THP-1 macrophages with or without IONPs-PAA-PEG-MAN, Rif@IONPs-PAA-PEG-MAN and rifampicin treatment. **C** Effects of IONPs-PAA- PEG-MAN, rifampicin and Rif@IONPs-PAA-PEG-MAN on the ratio of TNF-α + /IL-10 + in H37Rv-infected THP-1 macrophages, control group means H37Rv-infected THP-1 macrophages without drug treatment, n = 3, ANOVA-Tukey analysis was applied for the comparative analysis of data, **p < 0.01

In Mtb infected macrophages, M1 macrophages could release pro-inflammatory cytokines, such as TNF-α and IL-12, to trigger pro-inflammatory innate immunity for killing of intracellular Mtb, while M2 macrophages could release some anti-inflammatory cytokines, such as IL-10 and TGF-β, to inhibit host inflammatory responses against Mtb [25, 56]. Some host defense mechanisms of human alveolar macrophage against Mtb were found to be TNF-α-dependent, which could be impaired by virulent Mtb using IL-10 as an upstream mediator [56, 58]. These results thus highlighted the important roles of TNF-α/IL-10 axis in the host cell defense mechanisms against Mtb. To understand how IONPs-PAA-PEG-MAN induced M1 macrophage polarization contributes to intracellular Mtb clearance, we further tested the intracellular TNF-α and IL-10 levels in H37Rv-infected THP-1 macrophages. Our results demonstrated that IONPs-PAA-PEG-MAN could dramatically increase intracellular TNF-α level and decrease intracellular IL-10 level in Mtb infected THP-1 macrophages. Similarly, Rif@IONPs-PAA-PEG-MAN could significantly increase TNF-α but decrease IL-10 in Mtb infected THP-1 macrophages, while rifampicin alone did not show such effects (Fig. 6A, B). These results further suggested that IONPs-PAA-PEG-MAN and Rif@IONPs-PAA-PEG-MAN could polarize Mtb infected macrophages into anti-microbial M1 phenotypes, up-regulate TNFα-induced anti-TB responses [59] by significantly increasing the TNF-α+/IL-10 + ratio (Fig. 6C), which might ultimately enhance anti-microbial innate immunity for Mtb killing.

### Intracellular concentration of rifampicin in Rif@IONPs-PAA-PEG-MAN nanodecoy treated macrophages

**Fig. 7:**
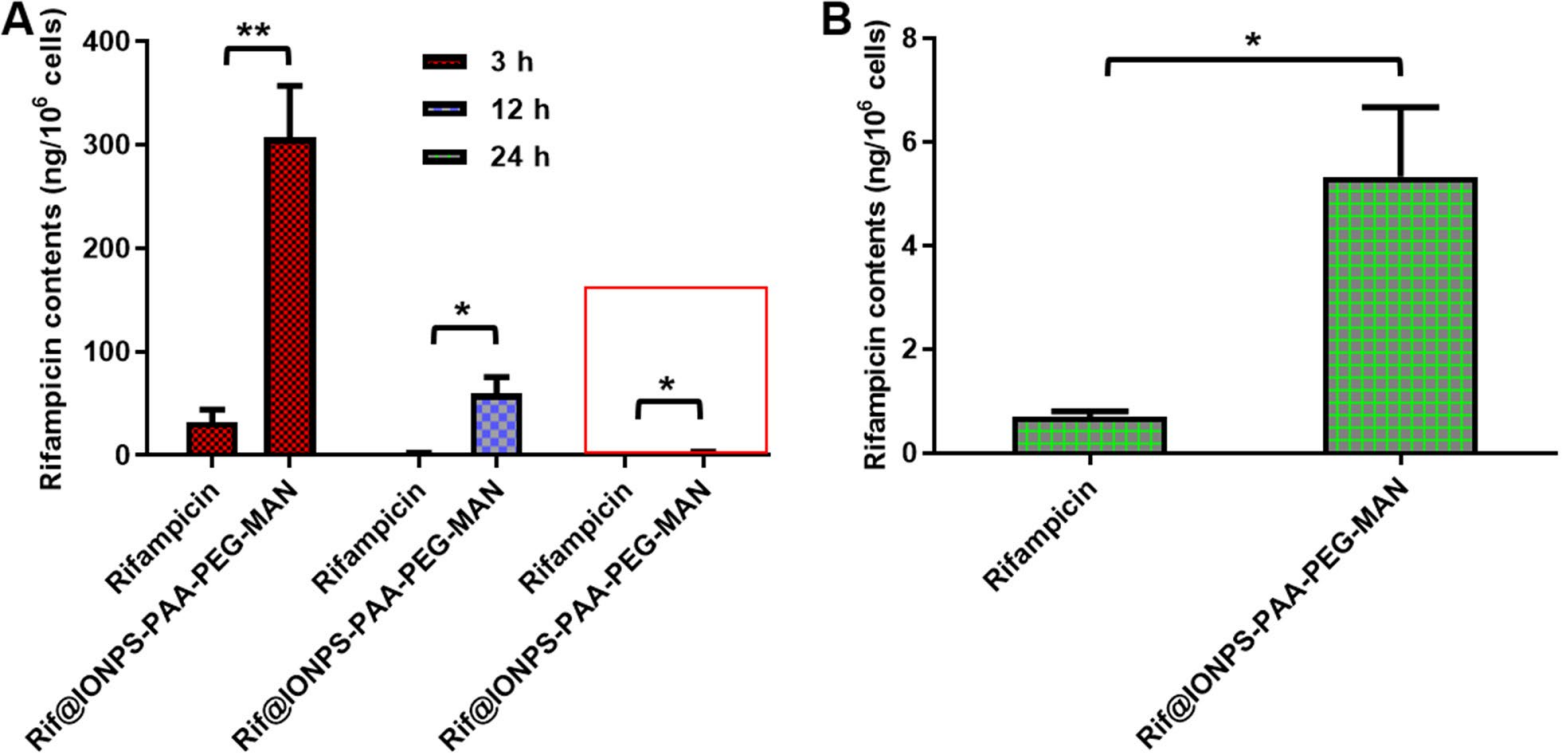
Intrcaellular rifampicin concentration in macrophages after rifampicin or Rif@IONPs-PAA-PEG-MAN treatment. **A** Intrcaellular rifampicin concentration in THP-1 cells after 3 h, 12 h and 24 h rifampicin or Rif@IONPs-PAA-PEG-MAN treatment, n = 3, multiple t-test analysis was applied for the comparative analysis of data, *p < 0.05, **p < 0.01. **B** Enlarged figure of the red box indicated in (**A**) for intracellular rifampicin concentration in THP-1 cells after 24 h rifampicin or Rif@IONPs-PAA-PEG-MAN treatment, n = 3, t-test analysis was applied for the comparative analysis of data, *p < 0.05

To explore the utility and unique potential of IONPs-PAA-PEG-MAN as a macrophage-targeted antibiotic delivery system for TB therapy, we quantified the intracellular rifampicin concentrations in THP-1 macrophages after treatment with Rif@IONPs-PAA-PEG-MAN or with same dosage of free rifampicin. As shown in Fig. 7, THP-1 macrophages treated with Rif@IONPs-PAA-PEG-MAN showed much and significantly higher intracellular rifampicin contents than that of free rifampicin-treated cells. Of note, the time-dependent decreasing trend of intracellular rifampicin contents in both settings (Fig. 7) might be attributed to the ability of macrophages to decrease intracellular antibiotic contents by the multidrug resistance-associated transporters of eukaryotic cells [60, 61]. However, the macrophages treated with Rif@IONPs-PAA-PEG-MAN, but not free rifampicin, exhibited a significantly-delayed decrease in intracellular rifampicin concentration and maintained much higher intracellular rifampicin concentration at 12 and 24 h than that of control cells treated with free rifampicin (Fig. 7). Such a delayed decrease of intracellular rifampicin might be associated with the controlled release property of rifampicin by Rif@IONPs-PAA-PEG- MAN. Based on the ability to increase rifampicin uptake and sustain intracellular rifampicin contents, this macrophage-targeted Rif@IONPs-PAA-PEG-MAN system is expected to achieve better intracellular mycobactericidal effects against Mtb.

### Effects of Rif@IONPs-PAA-PEG-MAN nanodecoy on bacterial burden in Mtb infected macrophages

Our above results already demonstrated that the macrophage-targeted Rif@IONPs-PAA-PEG-MAN could function as drug loading/delivery system to specifically increase the cellular uptake of rifampicin and sustain high intracellular contents, while could also enhance anti-microbial innate immunity for potential synergistic intracellular Mtb killings. Additionally, Rif@IONPs-PAA-PEG-MAN could serve as a kind of novel nanodecoy for the “iron-tropic” Mtb to induce the very close localization of the lysosome accumulated Rif@IONPs-PAA-PEG-MAN surrounding Mtb localized phagosomes and the co-localization of Rif@IONPs-PAA-PEG-MAN with Mtb in lysosomes. Now, we were in a unique position to determine whether the macrophage-targeted Rif@IONPs-PAA-PEG-MAN nanodecoy could synergistically enhance the clearance of intracellular Mtb in Mtb infected macrophages. To this end, we comparatively tested killing efficiencies for both the extracellular Mtb in culture and *in vivo/ex vivo* intracellular Mtb in macrophages using defined amounts of rifampicin as control.

**Fig. 8:**
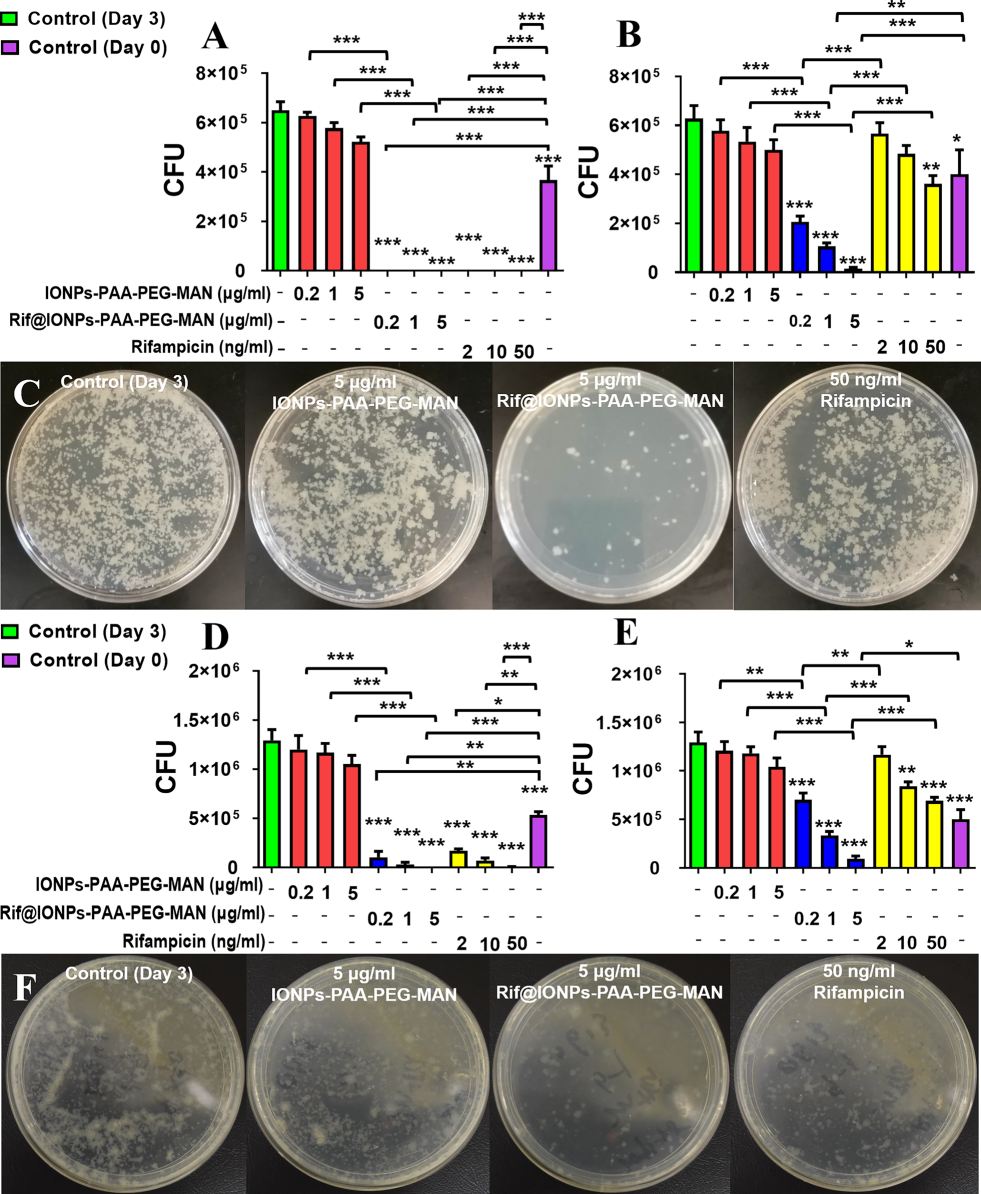
Enhanced killing efficiency of Rif@IONPs-PAA-PEG-MAN on intracellular M.tb in infected macrophages. **A** Effects of IONPs-PAA-PEG-MAN, rifampicin and Rif@IONPs-PAA-PEG-MAN on the growth of extracellular BCG, control group means extracellular BCG without drug treatment, n = 3, ANOVA-Tukey analysis was applied for the comparative analysis of data, ***p < 0.001. **B** Effects of IONPs-PAA-PEG-MAN, rifampicin and Rif@ IONPs-PAA-PEG-MAN on the growth of intracellular BCG in infected THP-1 cells, control group means BCG-infected THP-1 macrophages without drug treatment, n = 3, ANOVA-Tukey analysis was applied for the comparative analysis of data, ***p < 0.001. **C** Typical images of 7H11 plates for intracellular BCG in infected THP-1 cells, control group means BCG- infected THP-1 macrophages without drug treatment. **D** Effects of IONPs-PAA- PEG-MAN, rifampicin and Rif@IONPs-PAA-PEG-MAN on the growth of extracellular H37Rv, control group means extracellular H37Rv without drug treatment, n = 3, ANOVA-Tukey analysis was applied for the comparative analysis of data, ***p < 0.001. **E** Effects of IONPs-PAA-PEG-MAN, rifampicin and Rif@IONPs-PAA-PEG-MAN on the growth of intracellular H37Rv in infected THP-1 cells, control group means H37Rv-infected THP-1 macrophages without drug treatment, n = 3, ANOVA-Tukey analysis was applied for the comparative analysis of data, ***p < 0.001. **F** Typical images of 7H11 plates for intracellular H37Rv in infected THP-1 cells, control group means H37Rv-infected THP-1 macrophages without drug treatment

In testing extracellular Mtb in culture, we could clearly find that free rifampicin and Rif@IONPs-PAA-PEG-MAN could both significantly killed extracellular Mtb when compared both with the day0 control and day3 control (Fig. 8A, D). We also found that Rif@IONPs-PAA-PEG-MAN slightly enhanced the killing effects against extracellular BCG and H37Rv (Fig. 8A, D), which might be attributed to the regulation of drug efflux pumps of Mtb by IONPs for enhanced drug killing efficiency [55]. As shown in Fig. 8B, E, we found that IONPs-PAA-PEG-MAN showed subtle inhibition effects on intracellular BCG and H37Rv growth whereas high concentration of rifampicin alone could significantly inhibit intracellular BCG and H37Rv growth. However, Rif@IONPs-PAA-PEG-MAN significantly enhanced inhibition/killing effects against intracellular BCG and H37Rv when compared with the same dosages of IONPs-PAA-PEG-MAN and rifampicin. The significant decreases of CFU in 7H11 plates both for intracellular BCG (Fig. 8C) and intracellular H37Rv (Fig. 8F) were also found in Rif@IONPs-PAA-PEG-MAN treated THP-1 cells when compared to the same dosages of IONPs-PAA-PEG-MAN and rifampicin controls in the 3 days treatment setting. More importantly, using day 0 as initial bacilli level as control, we found that Rif@IONPs-PAA-PEG-MAN showed extremely lower CFU of intracellular Mtb than the control rifampicin alone, especially in high dosage groups, further indicating the synergistic killing effects of Rif@IONPs-PAA-PEG-MAN against intracellular Mtb in vitro.

**Fig. 9:**
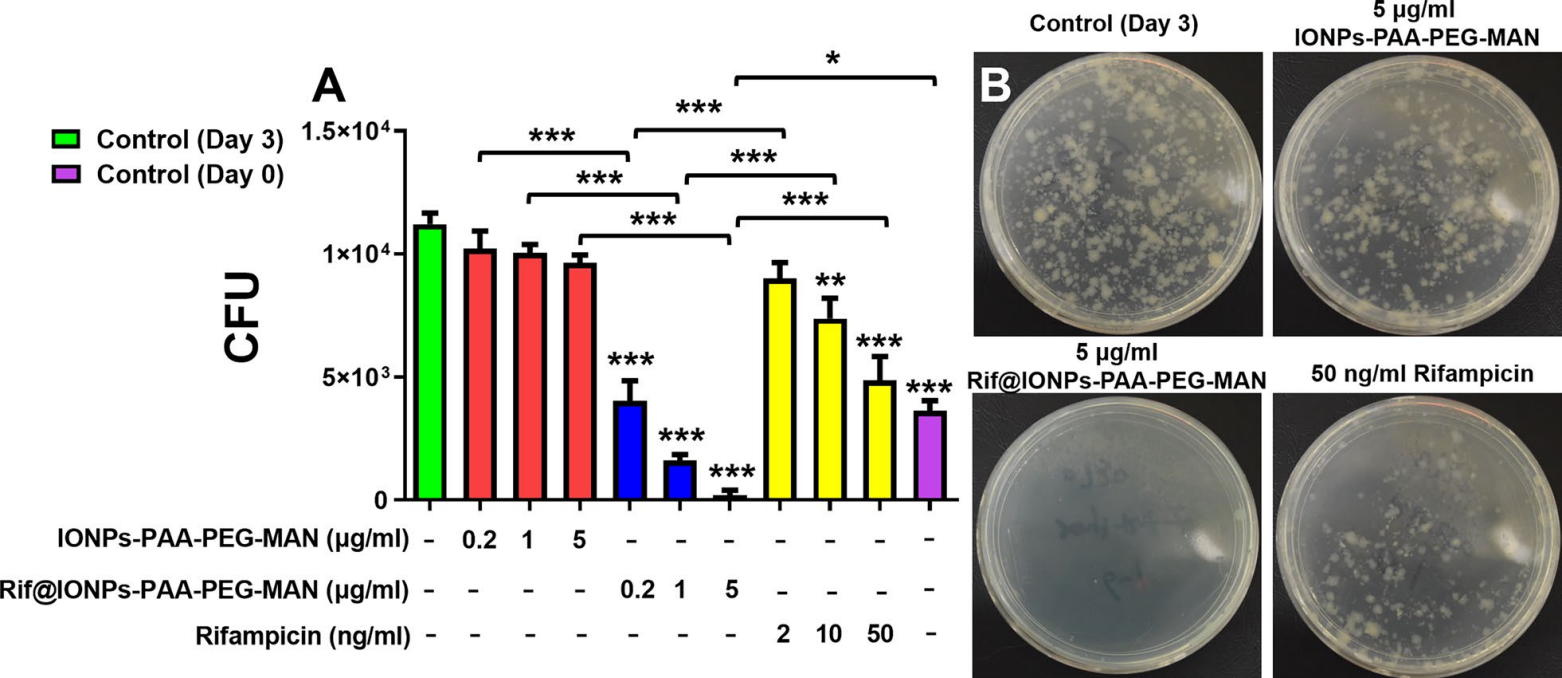
Enhanced killing efficiency of Rif@IONPs-PAA-PEG-MAN on intracellular H37Rv in monocyte-derived macrophages from rhesus macaques. **A** Effects of IONPs-PAA- PEG-MAN, rifampicin and Rif@IONPs-PAA-PEG-MAN on the growth of intracellular H37Rv in monocyte-derived macrophages from rhesus macaques, n = 3, ANOVA-Tukey analysis was applied for the comparative analysis of data, *p < 0.05, **p < 0.01, ***p < 0.001. **B** Typical images of 7H11 plates for intracellular H37Rv in monocyte-derived macrophages from rhesus macaques, control group means H37Rv-infected monocyte-derived macrophages without drug treatment

Since monocytes can readily differentiate into macrophages, we further tested killing effects of Rif@IONPs-PAA-PEG-MAN on intracellular Mtb in ex vivo monocytes isolated from healthy rhesus macaques. Consistently, Rif@IONPs-PAA-PEG-MAN significantly enhanced the killing effects of intracellular H37Rv in ex vivo monocytes, when compared to control rifampicin alone and control IONPs-PAA-PEG-MAN treatment (Fig. 9). And the high-concentration of Rif@IONPs-PAA-PEG-MAN indeed could synergistically kill the intracellular Mtb to a non-detectable or extremely low level of CFU counts when compared with the day0 control group and day3 control group (Fig. 9A, B). These results collectively demonstrated that the proposed Rif@IONPs-PAA-PEG-MAN nanodecoy could effectively combine both the innate immunity and the rifampicin killing efficacy for synergistic killings of intracellular Mtb ex vivo.

Based on these results above, we proposed a nanodecoy-enhanced anti-TB strategy based on Rif@IONPs-PAA-PEG-MAN for synergistic killings of intracellular Mtb by augmenting innate immunity killing and drug mycobactericidal effects in macrophages. Drug incorporated Rif@IONPs-PAA-PEG-MAN could be selectively internalized by Mtb infected macrophages through mannose receptor interaction, endocytosis, micropinocytosis, or phagocytosis, and then accumulate into lysosomes. In the intracellular compartments, Rif@IONPs-PAA-PEG-MAN could serve as a novel nanodecoy based on the iron tropism property of Mtb, which not only promote the localization of lysosome accumulated Rif@IONPs-PAA-PEG-MAN surrounding Mtb hided phagosomes, but also induce the direct co-localization of Rif@IONPs-PAA-PEG-MAN and Mtb within lysosomes. The direct Mtb exposure to the large amounts of rifampicin and potentially excessive iron released from Rif@IONPs-PAA-PEG-MAN would significantly kill the intracellular Mtb. Concurrently, Rif@IONPs-PAA-PEG-MAN could also augment broader innate immunity killing of intracellular Mtb by promoting M1 anti-microbial polarization of the Mtb infected macrophages and increasing the production of anti-TB cytokine TNF-α, while reducing the antagonizing cytokine IL-10. This novel nanodecoy-enhanced anti-TB strategy combining broader innate immunity killings and antibiotic mycobactericidal efficiencies is expected to serve as more efficient treatments for synergistic clearance of Mtb infection.

### **Effects Rif@IONPs-PAA-PEG-MAN nanodecoy on **in vivo **mycobacterial burden and bacilli-driven inflammation in Mtb infected mice**

**Fig. 10:**
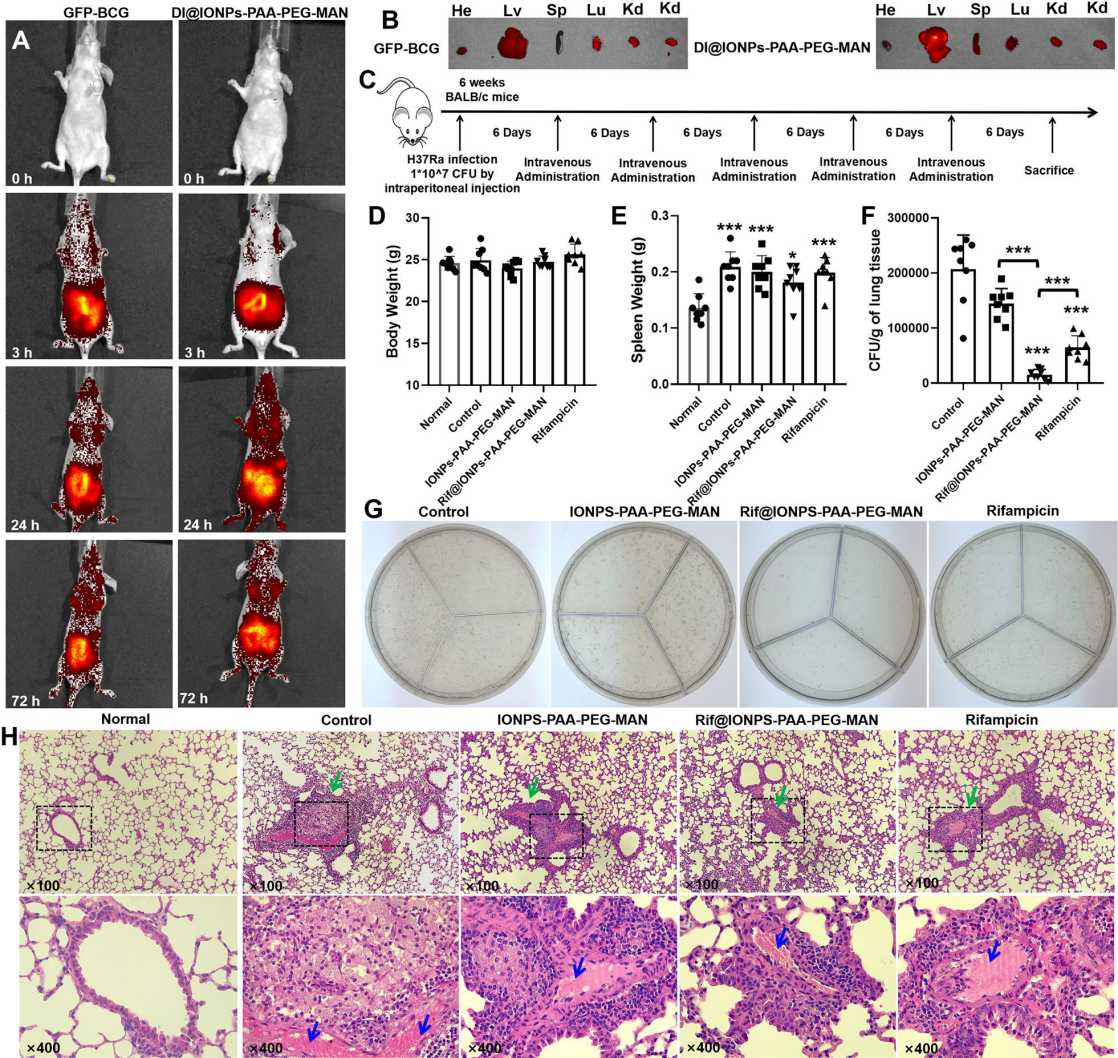
In vivo anti-TB effects of Rif@IONPs-PAA-PEG-MAN nanodecoy-assisted anti-TB strategy in Mtb infected mice. **A** IVIS imaging for in vivo distribution of GFP-BCG and DI@IONPs-PAA-PEG-MAN in mice at different time points after intravenous injection. **B** IVIS imaging for the distribution of GFP-BCG and DI@IONPs-PAA-PEG-MAN in different organs of mice after 72 h of intravenous injection (sacrificed), He means heart, Lv means liver, Sp means spleen, Lu means lung and Kd means kidney. **C** Diagram for the exprimental design of Mtb infected mice model and drug administration for the Mtb infected mice, normal group means mice without Mtb infection and drug treatment, control group means mice with Mtb infection and with saline treatment. **D** Body weight of mice in different groups at the endpoint of the experiment, n = 8. **E** Spleen weight of mice in different groups at the endpoint of the experiment, n = 8, ANOVA-Tukey analysis was applied for the comparative analysis of data, *p < 0.05, ***p < 0.001. **F** Bacterial loads in the lung of mice from Mtb infected mice at the endpoint of the experiment, n = 8, ANOVA-Tukey analysis was applied for the comparative analysis of data, ***p < 0.001. **G** Typical 7H11 plates coated with the lysis of lungs for CFU counting of Mtb in the lungs at the endpoint of the experiment. **H** Typical images of the H&E stained lung tissues of mice from different groups, green arrows indicated the inflammatory infiltrates and hemorrhages structures and blue arrows indicated the red cells

Based on the in vitro and ex vivo anti-mycobacterial activities of Rif@IONPs-PAA-PEG-MAN nanodecoy, it’s necessary to further understand the in vivo effects of these NP for potential anti-TB application. Firstly, we examined the in vivo and ex vivo distribution of mycobacteria and IONPs-PAA-PEG-MAN in nude mice after intravenous injection using GFP-BCG as mycobacterial infection model. The whole-body fluorescence imaging showed that GFP-BCG was mainly distributed in the enterocoeles and chest after intravenous injection through tail vein, while DI@IONPs-PAA-PEG-MAN showed similar distributions (Fig. 10A). In the imaging of *ex vivo mouse* organs, we could clearly see that most GFP-BCG and DI@IONPs-PAA-PEG-MAN were mainly distributed in the liver and lung of mice after 72 h of intravenous injection through tail vein (Fig. 10B). Thus, the in vivo distribution trend of IONPs-PAA-PEG-MAN nano-system and BCG appeared to be relevant to delivery of our NP to potential Mtb infection, suggesting that our NP could serve as potential drug carrier for in vivo anti-mycobacterial treatments.

To further explore our NP-mediated in vivo anti-mycobacterial/bactericidal effects, we utilized Mtb-H37Ra-infected mice as acute infection model to assess Rif@IONPs-PAA-PEG-MAN for potential therapeutics against Mtb infection and the associated inflammation (Fig. 10C). As shown in Fig. 10D, no significant body weight changes were seen in mice undergoing Mtb-H37Ra infection or drug treatments with (IONPs-PAA-PEG-MAN, Rif@IONPs-PAA-PEG-MAN or rifampicin. But we could observe significant increases in spleen weight of Mtb-H37Ra-infected mice compared to the uninfected normal mice, while Rif@IONPs-PAA-PEG-MAN treatment could partially improve the spleen weight changes after H37Ra infection (Fig. 10E). Through measuring CFU counts in the lung tissue lysates, we found that both rifampicin and Rif@IONPs-PAA-PEG-MAN treatment could significantly reduce the Mtb burdens in the lung of infected mice when compared with the control mice with Mtb infection (Fig. 10F). However, mice treated with Rif@IONPs-PAA- PEG-MAN showed significantly lower Mtb-H37Ra burdens (CFU counts) in the lung than those animals treated with IONPs-PAA-PEG-MAN or rifampicin-alone (Fig. 10F, G). These results from acute Mtb-H37Ra infection mouse model were consistent with the in vitro observation that rifampicin-loaded Rif@IONPs-PAA-PEG-MAN could facilitate delivery of rifampicin into infected macrophages/phagosomes and enhance rifampicin bactericidal killing of intracellular Mtb bacilli.

The typical H&E staining of lung tissues indicated that short-term Mtb-H37Ra infection of mice could induce mycobacterium-driven inflammation, characterized by inflammatory infiltrates and hemorrhages in certain lung tissues (Fig. 10H). In contrast, Rif@IONPs- PAA-PEG-MAN treatment of Mtb-H37Ra-infected mice could partially alleviate the H37Ra-driven inflammation in the lung (Fig. 10I, middle-right). These results suggested that Rif@IONPs-PAA-PEG-MAN treatment could also significantly reduce the Mtb-H37Ra burdens and the associated inflammation in the lung of infected mice.

**Fig. 11:**
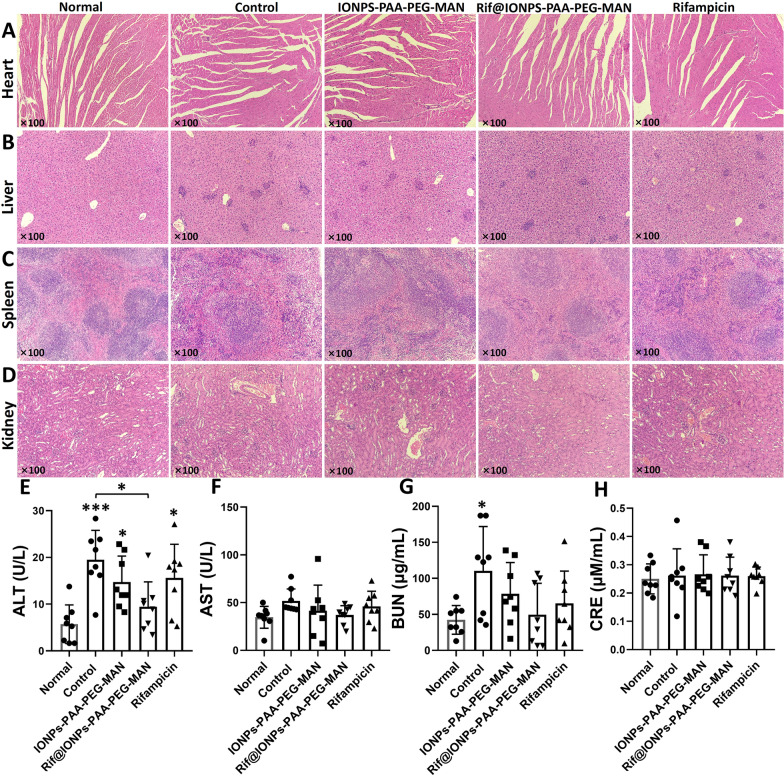
Organ tissue structures and serum hepatic/renal function evaluations of mice, normal groups means mice without Mtb infection and drug treatment, control group means mice with Mtb infection and with saline treatment. **A** Typical images of the H&E stained heart tissues of mice from different groups. **B** Typical images of the H&E stained liver tissues of mice from different groups. **C** Typical images of the H&E stained spleen tissues of mice from different groups. **D** Typical images of the H&E stained kidney tissues of mice from different groups. **E** Serum ALT concentrations in mice from different groups, n = 8, *p < 0.05, ANOVA-Tukey analysis was applied for the comparative analysis of data, ***p < 0.001. **F** Serum AST concentrations in mice from different groups, n = 8. **G** Serum BUN concentrations in mice from different groups, n = 8, ANOVA-Tukey analysis was applied for the comparative analysis of data, *p < 0.05. **H** Serum CRE concentrations in mice from different groups, n = 8

Furthermore, we sought to determine whether Rif@IONPs-PAA-PEG-MAN treatment could enhance rifampicin killing of Mtb-H37Ra and attenuate the mycobacterium-driven inflammation and injury in other organs (heart, liver, spleen and kidney) of the infected mice. In this context, we also performed initial assessment of potential toxicity of the NP treatment, as those organs usually harbor circulating/disseminating mycobacteria and NP. While Mtb-H37Ra infection led to mycobacterium-driven inflammation in heart, liver, spleen and kidney tissues, Rif@IONPs-PAA-PEG-MAN treatment of infected mice could partially alleviate these changes without remarkable tissue toxicity (Fig. 11A–D). Furthermore, we measured serum levels of glutamic oxalacetic transaminase (AST), glutamic-pyruvic transaminase (ALT) and blood urea nitrogen (BUN), and creatinine (CRE) as “liver/kidney function” parameters to examine acute injury as routinely done in clinic. While Mtb-H37Ra infection significantly increased the ALT level when compared with that of normal mice, Rif@IONPs-PAA-PEG-MAN treatment significantly reversed the Mtb-H37Ra-driven increases in ALT in serum to a level similar with the normal mice without Mtb infection (Fig. 11E). However, neither Mtb-H37Ra infection nor Rif@IONPs-PAA-PEG-MAN treatment led to any significant increases in other parameters except for Mtb-H37Ra-driven elevation of BUN alone (Fig. 11F–H).

Thus, the above results indicated that H37Ra acute infection mouse model allowed us to confirm our concept as proven in the cellular/molecular studies in the tissue culture setting. Together, the rifampicin-loaded Rif@IONPs-PAA-PEG-MAN can better deliver rifampicin into macrophages/phagosomes, more efficiently kill intracellular mycobacteria and reduce infection levels/inflammation when compared with the control rifampicin alone. Data also implicate a safety profile for our NP, without detectable toxicity.

Based on these results above, we proposed a nanodecoy-enhanced anti-TB strategy based on Rif@IONPs-PAA-PEG-MAN for synergistic killings of intracellular Mtb by augmenting innate immunity killing and drug mycobactericidal effects in macrophages (Additional file 1: Fig. S9). Drug incorporated Rif@IONPs-PAA-PEG-MAN could be selectively internalized by Mtb-infected macrophages through mannose receptor interaction, endocytosis, micropinocytosis, or phagocytosis, and then accumulate into lysosomes. In the intracellular compartments, Rif@IONPs-PAA-PEG-MAN could serve as a novel nanodecoy based on the iron tropism property of Mtb, which not only promote the localization of lysosome accumulated Rif@IONPs-PAA-PEG-MAN surrounding Mtb hided phagosomes, but also induce the direct co-localization of Rif@IONPs-PAA-PEG-MAN and Mtb within lysosomes. The direct Mtb exposure to the large amounts of rifampicin and potentially excessive iron released from Rif@IONPs-PAA-PEG-MAN would significantly kill the intracellular Mtb. Concurrently, Rif@IONPs-PAA-PEG-MAN nanodecoy could also augment broader innate immunological killing/inhibition of intracellular Mtb by promoting M1 anti-microbial polarization of the Mtb infected macrophages and increasing the production of typical pro-inflammatroy/anti-TB cytokine associated with M1 macrophages, such as TNF-α, which were responsible for triggering the innate immunological responses against intracellular Mtb. Thus, this novel nanodecoy-enhanced anti-TB strategy combining broader innate immunity killings and antibiotic mycobactericidal efficiencies can serve as more efficient treatments for synergistic clearance of Mtb infection in vivo.

We agree that inhalation-based strategies can provide better patient compliance, convenience and long-term treatment adherence against the chronic conditions of lung airway/mucosal inflammation compared with invasive intravenous administration. In fact, the inhalation drug delivery in humans proves to be particularly useful for delivering those drugs to relax bronchoconstriction/airway narrowing in the setting of chronic asthma attacks. However, TB involves not only airway/mucosa inflammation, but also lung tissues/parenchyma with necrosis/tissue damage-fibrosis, which may make it difficult for drugs to be delivered to lung tissues via inhalation. The drugs in blood systems are expected to reach these tissues/parenchyma with necrosis/tissue damage-fibrosis more effectively. These aspects may partially explain why clinical anti-TB drugs (antibiotics) are often given by oral administration or needle-injection. In the current proof-of-concept study, we elected the intravenous route because our initial goal was to determine whether the traditional systemic administration (Oral administration was not used due to the acidic environment of gastric juice) enables the drug-loaded nanoparticles to be delivered to infected lungs with de novo macrophage innate immunity and drug-bactericidal effects. On the other hand, technical issues did not allow us to employ inhalation-based administration of drug to mice because mice cannot actively inhale the desired amounts of drugs as humans do. At this point, we and many other labs do not have expensive inhalation/aerosol equipment required for precise inhalation delivery of drugs. And intravenous injection would be easier and scientifically sound in mice compared with the respiratory tubing technique, which may cause off-target delivery due to potential tissue damage/drug leakage or spurt out. Therefore, in the current study, we chose intravenous route for IONPs-PEG-PAA-MAN administration for the treatment of infected mice. However, we will consider exploring inhalation-based strategies for in vivo anti-TB drug delivery in the future.

Moreover, the future application of macrophage-targeted nanosystems for anti-TB treatment still restricted by the physical obstruction of TB granulomas, which are complex hallmark structures of TB that form in lungs, composed of different immune cells surrounding bacteria infected cells (such as macrophages) and a caseous necrotic core. In theory, the formation of granulomas would limit the effectiveness of macrophage-targeted therapies (such as mannosylated nanoparticles), as the particles might not reach the bacteria infected macrophages effectively. Thus, although mannosylated nanosystem are developed for anti-TB treatment, more works are needed in the future to overcome the difficulties about how to penetrate into the granuloma structures to reach the Mtb infected macrophages with high efficiency, which may be critical for the future application of mannosylated nanosystem for clinical anti-TB treatment.

## Conclusions

To our knowledge, for the first time this work introduces macrophage-targeted iron oxide nanoparticles as a kind of poisonous nanodecoy for the “iron-tropic” Mtb to enhance drug uptake/accumulation in macrophages, which promote the accumulation of drug loaded nanodecoy surrounding the Mtb hided phagosome or co-localization of drug loaded nanodecoy with Mtb in lysosomes. Such direct exposure of this Rif@IONPs-PAA-PEG- MAN nanodecoy to Mtb provides the convenience for synergistic killings of intracellular Mtb by manipulating augmented innate immunity and drug killing effects. Rif@IONPs-PAA- PEG-MAN demonstrated high biocompatibility with low cytotoxicity, and exhibited preferential uptake by Mtb infected macrophages through mannose receptor interaction, endocytosis, micropinocytosis, and phagocytosis. Intracellular Rif@IONPs-PAA-PEG-MAN mostly retained in acidic lysosomes, where rifampicin could be readily released from Rif@IONPs-PAA-PEG-MAN in acidic pH condition. The enhanced uptake and accumulation of rifampicin in macrophages were consistent with the sustainable high intracellular rifampicin contents than that of free rifampicin treated cells. Moreover, Rif@IONPs-PAA- PEG-MAN nanodecoy could also polarize Mtb infected macrophages into anti-mycobacterial M1 phenotypes and increase the M1 macrophage associated pro-inflammatory cytokine (TNF-α) production to trigger innate immunological responses for the killing and inhibition of intracellular Mtb. Collectively, Rif@IONPs-PAA-PEG-MAN could synergistically enhance the killings/clearance of intracellular Mtb, reduce the mycobacterial burdens in the lungs and alleviate the mycobacterium-driven inflammation in infected mice. In summary, the macrophage-targeted Rif@IONPs-PAA-PEG-MAN nanodecoy may potentiate better therapeutic strategy against TB and drug-resistant TB.

### Supplementary Information

**Additional file 1: Fig. S1. **TEM elemental mapping analysis of (A) IONPs-PAA-PEG-MAN and (B) Rif@IONPs-PAA-PEG-MAN, scale bar: 50 nm. **Fig. S2.** X-ray diffraction (XRD) analysis of (A) IONPs-PAA- PEG-MAN and (B) Rif@IONPs-PAA-PEG-MAN. **Fig. S3.** X-ray photoelectron spectroscopy (XPS) analysis of Fe 2p spectrum for (A) IONPs-PAA-PEG-MAN and (B) Rif@IONPs-PAA-PEG- MAN. **Fig. S4.** Effects of IONPs-PAA-PEG-MAN on the viability of THP-1 cells, RAW264.7 cells, Hlmvec cells and A549 cells, n=3. **Fig. S5.** Dose-dependent cellular uptake of C6@IONPs-PAA-PEG and C6@IONPs-PAA-PEG-MAN in THP-1 cells after (A) 0.5 h, (B) 1 h and (C) 3h treatment, n=3, *p<0.05, **p<0.01,***p<0.001. Dose- dependent cellular uptake of C6@IONPs-PAA-PEG-MAN in THP-1 cells and HLMVEC cells after (D) 0.5 h, (E) 1 h and (F) 3h treatment, n=3,*p<0.05,  ***p<0.001. **Fig. S6.** Fluorescence imaging for localization of GFP-BCG, DI@IONPs-PAA-PEG-MAN and lysosomes in THP-1 macrophages after (A) 6 h and (B) 24 h incubation, white arrow indicates the GFP-BCG located in lysosomes but not co-localized with DI@IONPs-PAA-PEG-MAN, yellow arrow indicates the GFP-BCG co-localized with DI@ IONPs-PAA-PEG-MAN in lysosomes and purple arrow indicates the GFP-BCG located outside lysosomes but co-localized with DI@IONPs-PAA-PEG-MAN. **Fig. S7.** H37Rv infected THP-1 macrophages after IONPs- PAA-PEG-MAN treatment (A-B) and Rif@IONPs-PAA-PEG-MAN treatment (C-D), H37Rv in phagosomes (indicated by yellow arrow) were surrounded by (A-B) IONPs-PAA-PEG-MAN in lysosomes (indicated by red arrow) or (C-D) Rif@ IONPs-PAA-PEG-MAN in lysosomes (indicated by blue arrow). **Fig. S8.** H37Rv infected THP-1 macrophages after Rif@IONPs-PAA-PEG-MAN treatment, H37Rv (indicated by white arrow) were fused into or located in lysosomes with Rif@IONPs-PAA-PEG-MAN (indicated by red arrow) inside. H37Rv in lysosomes were partially destroyed by Rif@IONPs- PAA-PEG-MAN into pieces to show very incompact and penetrable cross section morphology. **Fig. S9.** Proposed mechanisms of Rif@IONPs-PAA-PEG- MAN nanodecoy-assisted anti-TB strategy for synergetic intracellular Mtb clearance and* in vivo *Mtb clearance by manipulating enhanced drug killing efficiency and boosted innate immunity in host cells. By Figdraw.

## Data Availability

The datasets used and/or analyzed in the current study are available from the corresponding author on reasonable request.
