# Supplementary material for: Macrophage targeted iron oxide nanodecoys augment innate immunological and drug killings for more effective Mycobacterium Tuberculosis clearance
Source: J Nanobiotechnology. 2023 Oct 10;21:369. doi: 10.1186/s12951-023-02103-x (PMC10563239; doi:10.1186/s12951-023-02103-x)
Supplement: Supplementary file 1 — Additional file 1: Fig. S1. TEM elemental mapping analysis of (A) IONPs-PAA-PEG-MAN and (B) Rif@IONPs-PAA-PEG-MAN, scale bar: 50 nm. Fig. S2. X-ray diffraction (XRD) analysis of (A) IONPs-PAA- PEG-MAN and (B) Rif@IONPs-PAA-PEG-MAN. Fig. S3. X-ray photoelectron spectroscopy (XPS) analysis of Fe 2p spectrum for (A) IONPs-PAA-PEG-MAN and (B) Rif@IONPs-PAA-PEG- MAN. Fig. S4. Effects of IONPs-PAA-PEG-MAN on the viability of THP-1 cells, RAW264.7 cells, Hlmvec cells and A549 cells, n=3. Fig. S5. Dose-dependent cellular uptake of C6@IONPs-PAA-PEG and C6@IONPs-PAA-PEG-MAN in THP-1 cells after (A) 0.5 h, (B) 1 h and (C) 3h treatment, n=3, *p<0.05, **p<0.01,***p<0.001. Dose- dependent cellular uptake of C6@IONPs-PAA-PEG-MAN in THP-1 cells and HLMVEC cells after (D) 0.5 h, (E) 1 h and (F) 3h treatment, n=3,*p<0.05, ***p<0.001. Fig. S6. Fluorescence imaging for localization of GFP-BCG, DI@IONPs-PAA-PEG-MAN and lysosomes in THP-1 macrophages after (A) 6 h and (B) 24 h incubation, white arrow indicates the GFP-BCG located in lysosomes but not co-localized with DI@IONPs-PAA-PEG-MAN, yellow arrow indicates the GFP-BCG co-localized with DI@ IONPs-PAA-PEG-MAN in lysosomes and purple arrow indicates the GFP-BCG located outside lysosomes but co-localized with DI@IONPs-PAA-PEG-MAN. Fig. S7. H37Rv infected THP-1 macrophages after IONPs- PAA-PEG-MAN treatment (A-B) and Rif@IONPs-PAA-PEG-MAN treatment (C-D), H37Rv in phagosomes (indicated by yellow arrow) were surrounded by (A-B) IONPs-PAA-PEG-MAN in lysosomes (indicated by red arrow) or (C-D) Rif@ IONPs-PAA-PEG-MAN in lysosomes (indicated by blue arrow). Fig. S8. H37Rv infected THP-1 macrophages after Rif@IONPs-PAA-PEG-MAN treatment, H37Rv (indicated by white arrow) were fused into or located in lysosomes with Rif@IONPs-PAA-PEG-MAN (indicated by red arrow) inside. H37Rv in lysosomes were partially destroyed by Rif@IONPs- PAA-PEG-MAN into pieces to show very incompact and penetrable cross section morphology. Fig. S9. Proposed mechanisms of [file 12951_2023_2103_MOESM1_ESM.docx]

**Macrophage Targeted Iron Oxide Nanodecoys Augment Innate Immunological and Drug Killings for More Effective Mycobacterium Tuberculosis Clearance**

Ling Shen^1#*^,Kangsheng Liao^#2,3,4^, Enzhuo Yang^1,5^, Fen Yang^2,3,4^, Wensen Lin^2,4^, Jiajun Wang^2,4^, Shuhao Fan^2,4^, Xueqin Huang^2^, Lingming Chen^2,3,4^, Hongbo Shen^5^, Hua Jin^2^, Yongdui Ruan^2^, Xing Liu^6^, Gucheng Zeng^7^, Jun-Fa Xu^2,4^*, Jiang Pi^2,3,4^#*

^1^ Department of Microbiology and Immunology, University of Illinois at Chicago, Chicago, IL, USA

^2^ Guangdong Provincial Key Laboratory of Medical Molecular Diagnostics, The First Dongguan Affiliated Hospital, Guangdong Medical University, Dongguan, China

^3^ The Marine Biomedical Research Institute, Guangdong Medical University, Zhanjiang, Guangdong, China; The Marine Biomedical Research Institute of Guangdong Zhanjiang, Guangdong, China

^4^ Institute of Laboratory Medicine, School of Medical Technology, Guangdong Medical University, Dongguan, China

^5^ Clinic and Research Center of Tuberculosis, Shanghai Key Lab of Tuberculosis, Shanghai Pulmonary Hospital, Tongji University School of Medicine, Shanghai, China

^6^ Key Laboratory of Animal Disease Diagnostics and Immunology, Ministry of Agriculture, MOE International Joint Collaborative Research Laboratory for Animal Health & Food Safety, College of Veterinary Medicine, Nanjing Agricultural University, Nanjing, China

^7^ Department of Microbiology, Zhongshan School of Medicine, Key Laboratory for Tropical Diseases Control of the Ministry of Education, Sun Yat-sen University, Guangzhou, Guangdong, China

^#^ These authors contribute equally and share the first authorship.

^*^ Corresponding authors

(L.S.): [lshen@uic.edu](mailto:lshen@uic.edu)

(J.X.): [xujunfa@gdmu.edu.cn](mailto:jiangpi@gdmu.edu.cn)

(J.P.): [jiangpi@gdmu.edu.cn](mailto:jiangpi@gdmu.edu.cn)


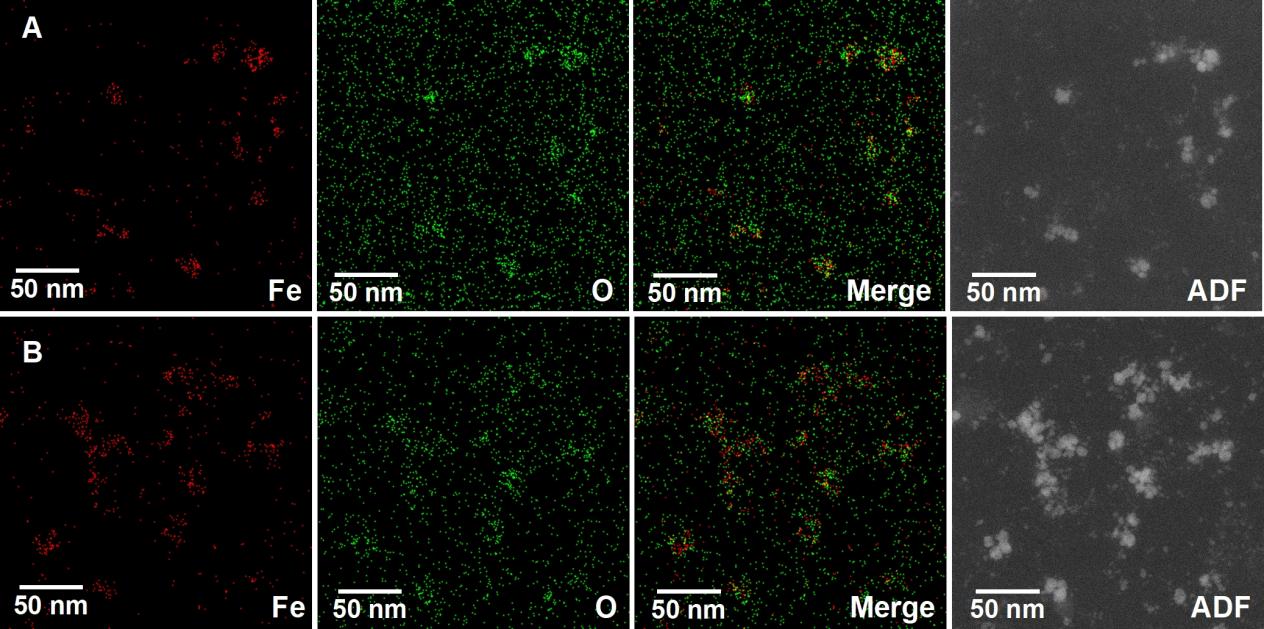


Additional file 1: Fig. S1 TEM elemental mapping analysis of (A) IONPs-PAA-PEG-MAN and (B) Rif@IONPs-PAA-PEG-MAN, scale bar: 50 nm.


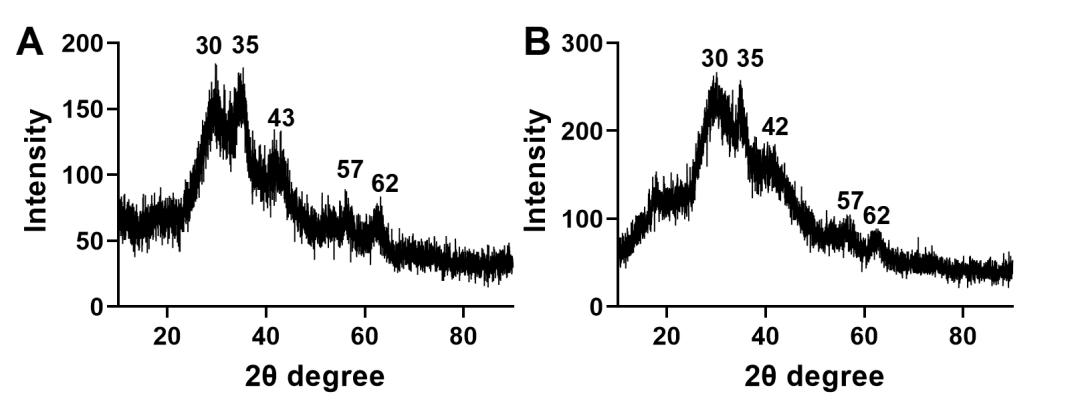


Additional file 1: Fig. S2 X-ray diffraction (XRD) analysis of (A) IONPs-PAA- PEG-MAN and (B) Rif@IONPs-PAA-PEG-MAN.


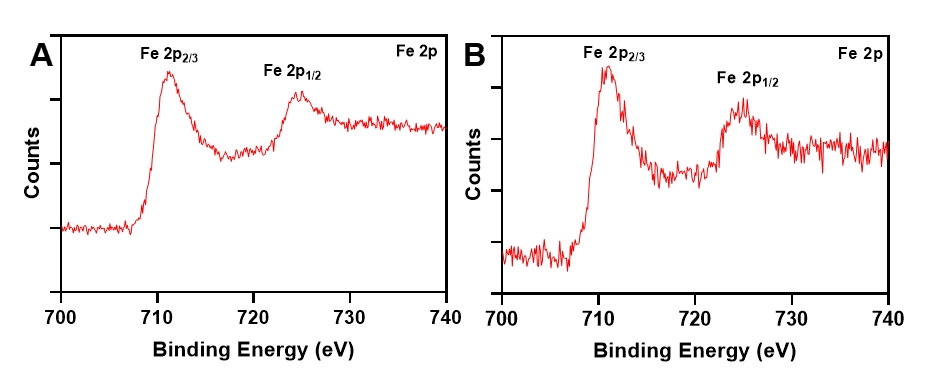


Additional file 1: Fig. S3 X-ray photoelectron spectroscopy (XPS) analysis of Fe 2p spectrum for (A) IONPs-PAA-PEG-MAN and (B) Rif@IONPs-PAA-PEG- MAN.


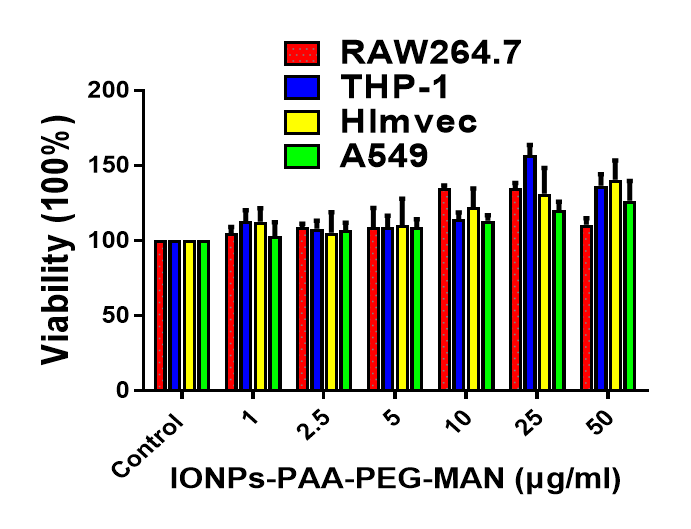


Additional file 1: Fig. S4 Effects of IONPs-PAA-PEG-MAN on the viability of THP-1 cells, RAW264.7 cells, Hlmvec cells and A549 cells, n=3.


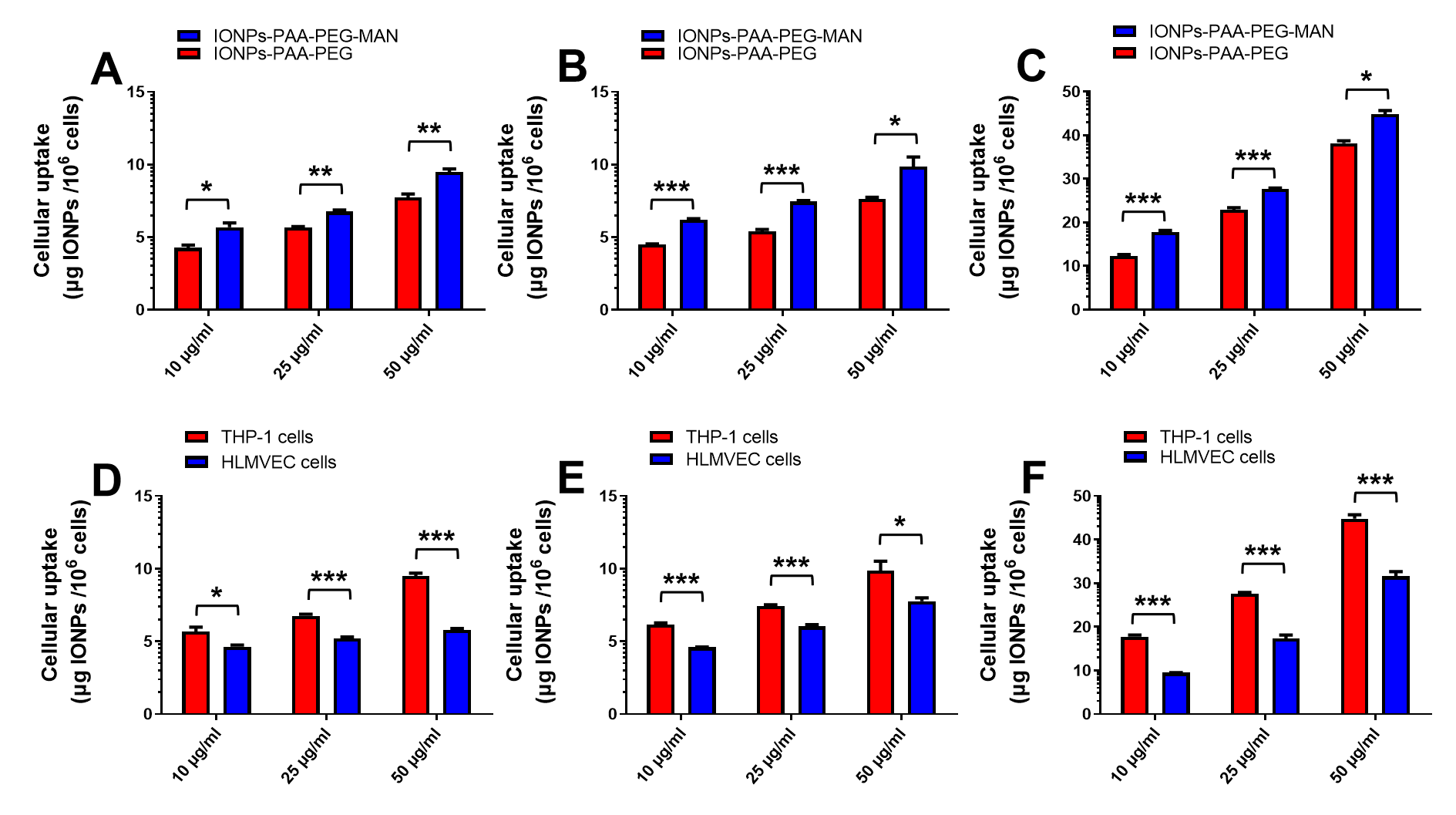


Additional file 1: Fig. S5 Dose-dependent cellular uptake of C6@IONPs-PAA-PEG and C6@IONPs-PAA-PEG-MAN in THP-1 cells after (A) 0.5 h, (B) 1 h and (C) 3h treatment, n=3, *p<0.05, **p<0.01, ***p<0.001. Dose- dependent cellular uptake of C6@IONPs-PAA-PEG-MAN in THP-1 cells and HLMVEC cells after (D) 0.5 h, (E) 1 h and (F) 3h treatment, n=3, *p<0.05, ***p<0.001.


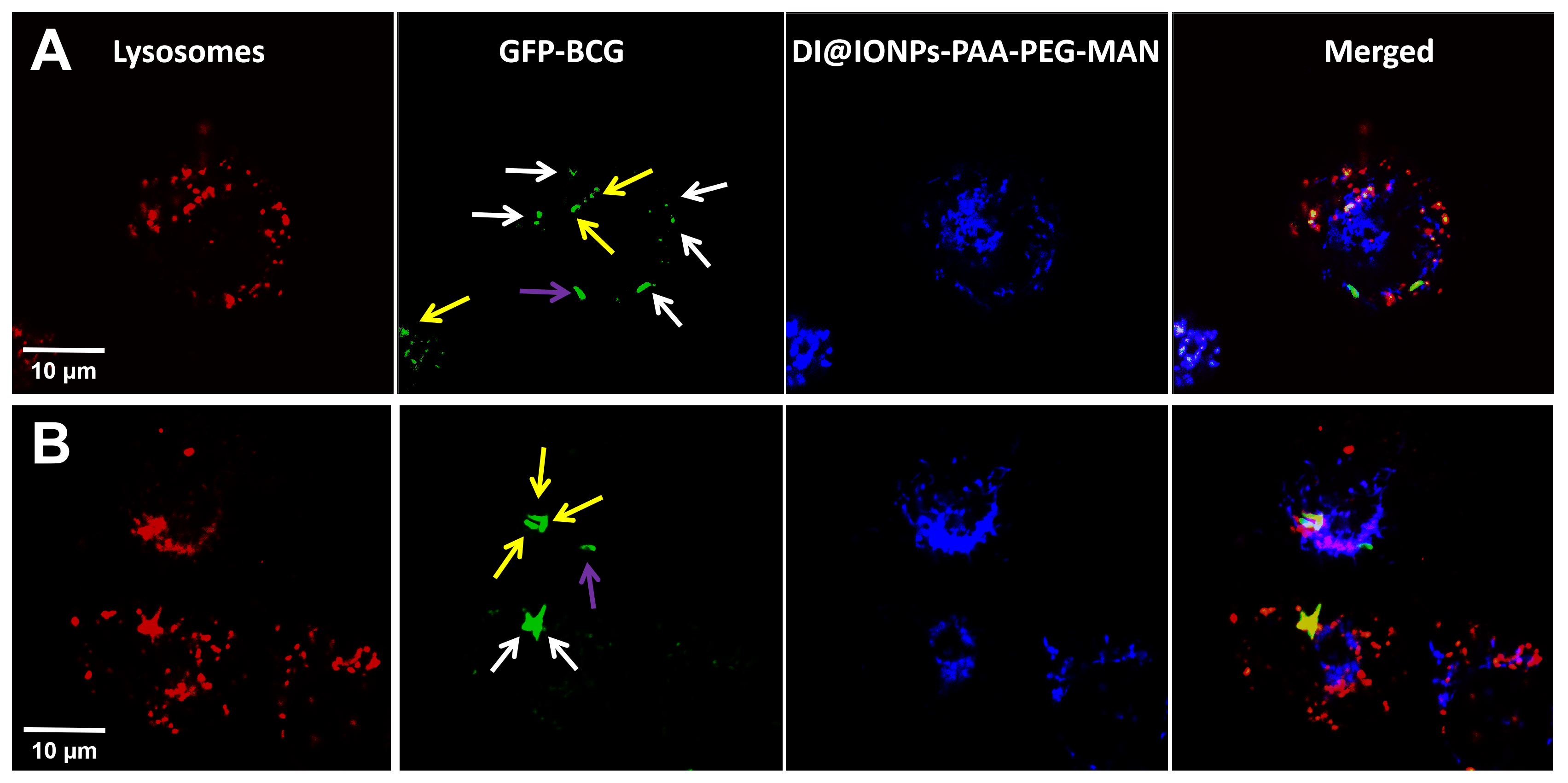


Additional file 1: Fig. S6 Fluorescence imaging for localization of GFP-BCG, DI@IONPs-PAA-PEG-MAN and lysosomes in THP-1 macrophages after (A) 6 h and (B) 24 h incubation, white arrow indicates the GFP-BCG located in lysosomes but not co-localized with DI@IONPs-PAA-PEG-MAN, yellow arrow indicates the GFP-BCG co-localized with DI@ IONPs-PAA-PEG-MAN in lysosomes and purple arrow indicates the GFP-BCG located outside lysosomes but co-localized with DI@IONPs-PAA-PEG-MAN.


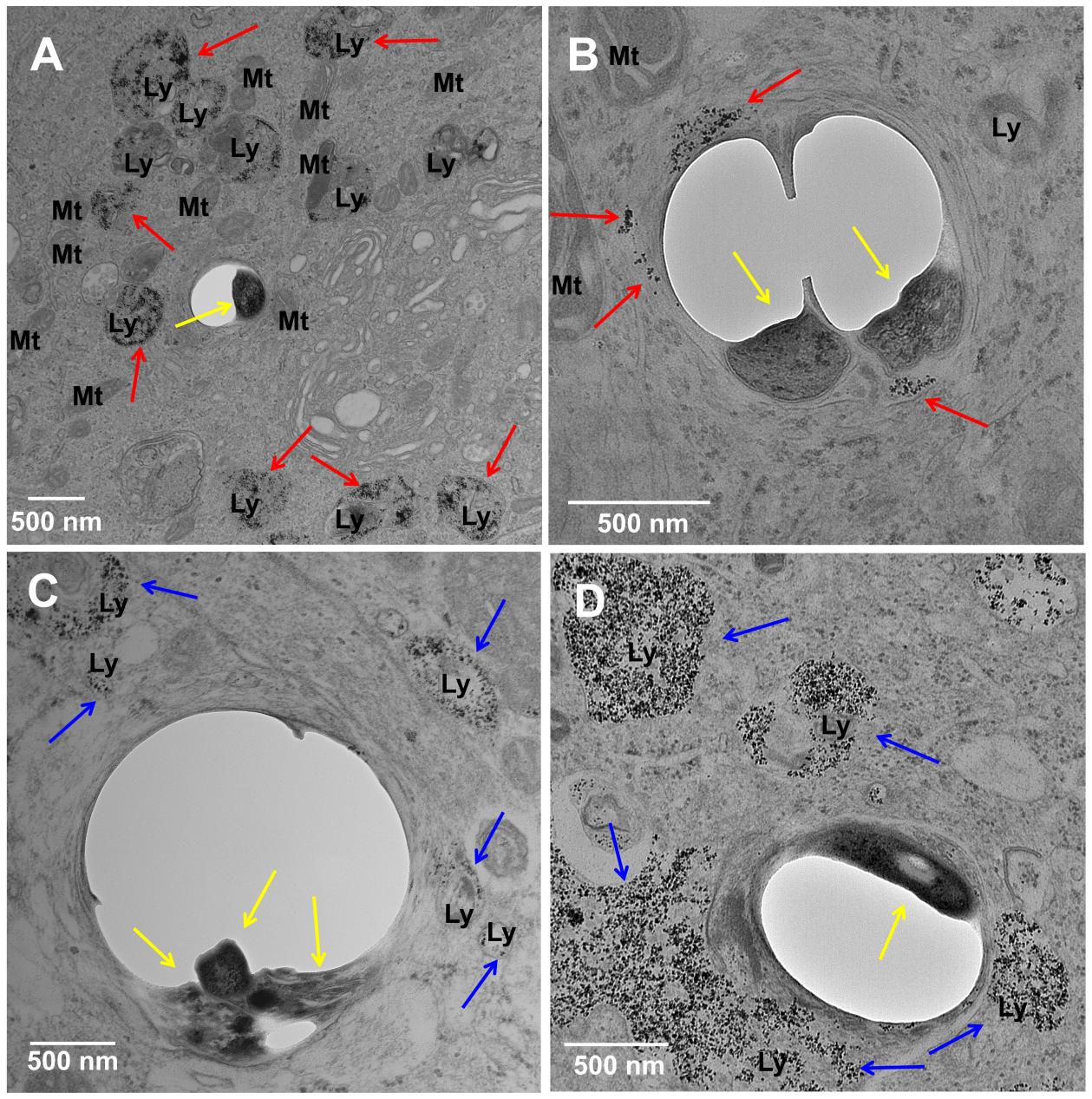


Additional file 1: Fig. S7 H37Rv infected THP-1 macrophages after IONPs- PAA-PEG-MAN treatment (A-B) and Rif@IONPs-PAA-PEG-MAN treatment (C-D), H37Rv in phagosomes (indicated by yellow arrow) were surrounded by (A-B) IONPs-PAA-PEG-MAN in lysosomes (indicated by red arrow) or (C-D) Rif@ IONPs-PAA-PEG-MAN in lysosomes (indicated by blue arrow).


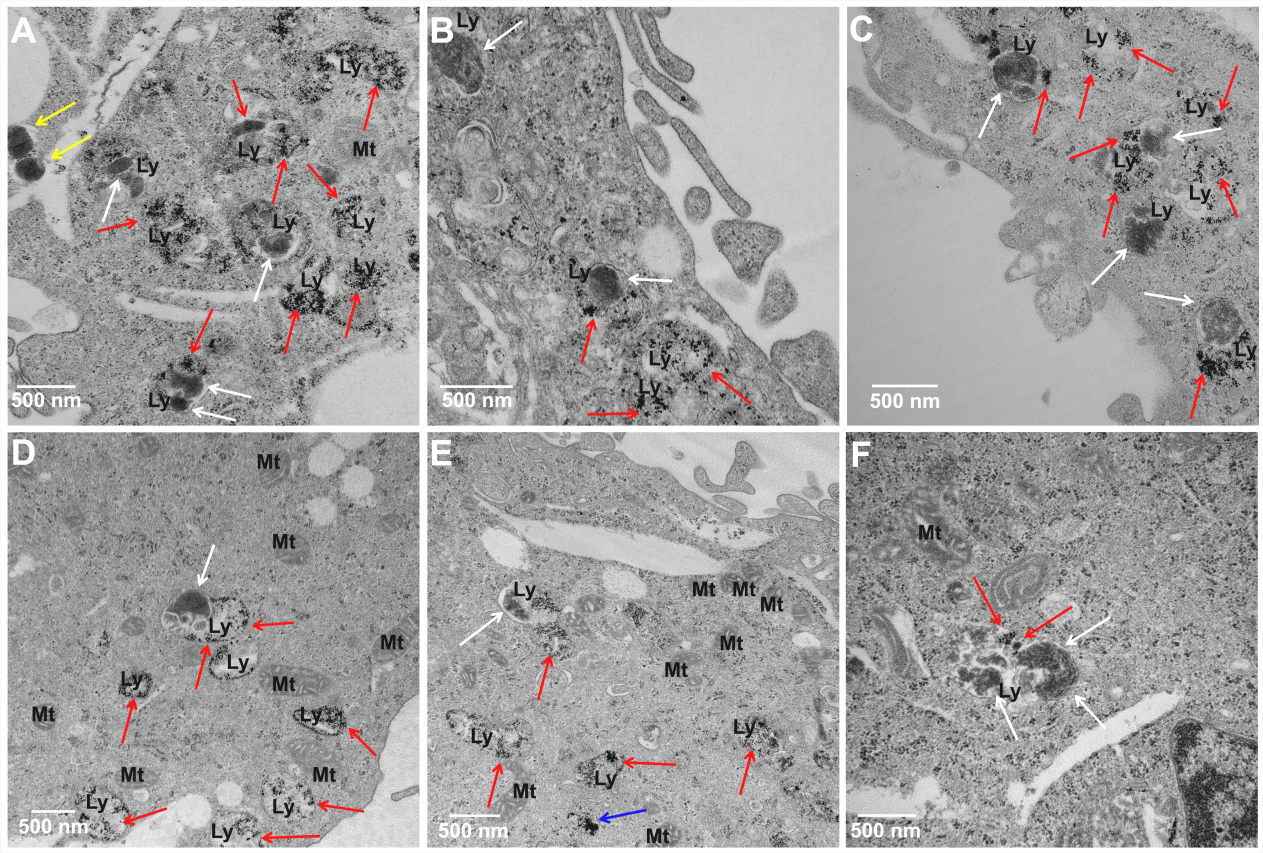


Additional file 1: Fig. S8 H37Rv infected THP-1 macrophages after Rif@IONPs-PAA-PEG-MAN treatment, H37Rv (indicated by white arrow) were fused into or located in lysosomes with Rif@IONPs-PAA-PEG-MAN (indicated by red arrow) inside. H37Rv in lysosomes were partially destroyed by Rif@IONPs- PAA-PEG-MAN into pieces to show very incompact and penetrable cross section morphology.


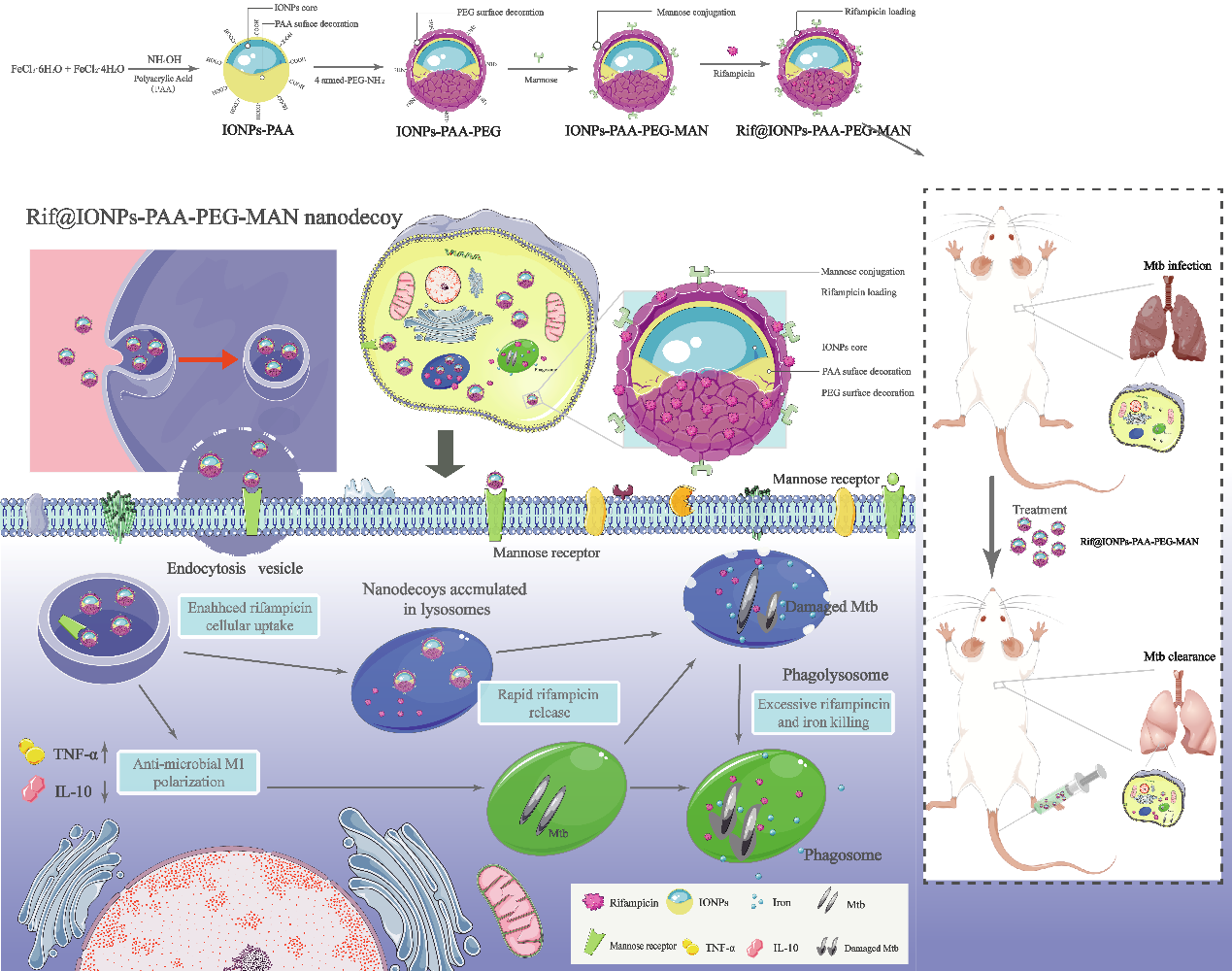


Additional file 1: Fig. S9 Proposed mechanisms of Rif@IONPs-PAA-PEG- MAN nanodecoy-assisted anti-TB strategy for synergetic intracellular Mtb clearance and *in vivo* Mtb clearance by manipulating enhanced drug killing efficiency and boosted innate immunity in host cells. By Figdraw.
